# Endothelial progenitor cell susceptibility to DNA damaging and DDR-modulating compounds determines endothelial differentiation accuracy

**DOI:** 10.1186/s13287-026-05087-1

**Published:** 2026-06-25

**Authors:** Sina Federmann, Michelle Westerhoff, Andreas S. Reichert, Gerhard Fritz

**Affiliations:** 1 Institute of Toxicology, Medical Faculty and University Hospital, Heinrich-Heine-University Duesseldorf, Moorenstrasse 5, 40225 Duesseldorf, Germany; 2 Institute of Biochemistry and Molecular Biology I, Medical Faculty and University Hospital, Heinrich-Heine-University Duesseldorf, Universitätsstrasse 1, 40225 Duesseldorf, Germany

**Keywords:** Murine embryonic stem cells (mESC), Endothelial differentiation, Anticancer therapy, Anthracyclines, DNA damage, DNA damage response (DDR), Cardiotoxicity, Regeneration

## Abstract

The clinical use of the anticancer drug doxorubicin (Dox) is limited by irreversible cardiotoxicity. The pathophysiological relevance of different cardiac cell types, including endothelial progenitor cells (EPC), in this process is unclear. Since progenitor cells are particularly relevant for tissue regeneration, we hypothesize that residual damage resulting from Dox-based therapeutic regimen may influence their endothelial differentiation accuracy. Therefore, we comparatively investigated the response of murine embryonic stem cells (mESC), endothelial progenitor cells (EC d4) and terminally differentiated endothelial-like cells (EC d6) following exposure to Dox and selected pharmacological inhibitors of DNA repair/DNA damage response (DDR) (RAD51i B02; HDACi entinostat (EST)). We show that EC d4 exhibit enhanced Dox sensitivity as compared to mESC and EC d6. EdU incorporation and replication fork progression analyses revealed pronounced agent-specific differences between mESC, EC d4 and EC d6. Furthermore, DNA damage formation varied in a drug-dependent manner, with mESC showing enhanced residual levels of DNA single-strand breaks (SSB) as compared to EC d4 and EC d6 while EC d6 revealed highest levels of DNA double-strand breaks (DSB). Dox treatment of EC d4 did not prevent their further differentiation into EC d6. However, it caused several functional impairments in the surviving EC d6 progeny, including defects in mitochondrial homeostasis, endothelial barrier function related to cell-cell adhesion factors (ZO1, VE-cadherin), cytokine response and low-density lipoprotein (LDL) uptake. This is accompanied by increased senescence. Summarizing, we demonstrate both overlapping and agent-specific responses of mESC, EC d4 and EC d6 to Dox and DNA repair/DDR inhibitors. Notably, drug treatment of EPC (EC d4) causes multiple dysfunctions in differentiated EC d6. Hence, pharmacological measures aiming to specifically protect EPC from Dox-induced damage are suggested to foster the maintenance of healthy endothelial functionality during regeneration, thereby lowering the risk of detrimental late cardiotoxicity resulting from Dox-based anticancer regimen.

**Supplementary Information:**

The online version contains supplementary material available at 10.1186/s13287-026-05087-1.

## Highlights

- EC d4 are most vulnerable towards Dox-induced cytotoxicity independent of drug transport.
- EC d4 and EC d6 display higher levels of drug-induced DSB as compared to mESC.
- Drug-induced replication stress is highest in mESC and decreases with differentiation.
- Both Dox and DNA repair/DDR inhibitors damage mitochondrial homeostasis.
- Differentiated EC d6 derived from drug-treated EC d4 display multiple functional impairments.

**Supplementary Information:**

The online version contains supplementary material available at 10.1186/s13287-026-05087-1.

## Introduction

Anthracycline derivatives such as doxorubicin (Dox) are widely used anticancer drugs [1], which act as type II topoisomerase (Topo II) poisons, thereby leading to the formation of highly cytotoxic DNA double-strand breaks (DSBs) [2]. DSBs are potent triggers of the DNA damage response (DDR), which regulates cell cycle progression, DNA repair and apoptosis, thereby defining the balance between survival and death-related mechanisms [3]. In addition, generation of reactive oxygen species (ROS), DNA intercalation, inhibition of DNA helicases and chromatin damage contribute to Dox-induced cytotoxicity [4, 5]. It is believed that at clinically relevant low Dox concentrations the inhibition of topoisomerase II (TopoII) isoforms and subsequent formation of DSB is particularly relevant for its cytotoxicity. By contrast, using higher Dox concentrations, oxidative stress and DNA intercalation likely become more relevant as toxic mode of action [6]. Cumulative and irreversible cardiotoxicity ultimately leading to cardiomyopathy and congestive heart failure is the clinically most relevant adverse effect of anthracyclines [1]. In view of the low antioxidative capacity of cardiomyocytes, mitochondria-related iron-dependent and iron-independent ROS formation may be of pathophysiological relevance for Dox-induced cardiac damage, with p53-regulated mechanisms of senescence and cell death being involved [7–11]. Hence, chemoprevention of anthracycline-mediated oxidative stress has been considered to achieve cardio-protection [10–12]. Yet, antioxidants failed to demonstrate substantial cardioprotective potency following anthracycline-based anticancer therapy [4, 13, 14]. By contrast, the EDTA analogue dexrazoxane, which is a strong catalytic inhibitor of topoisomerase II, is able to prevent anthracycline-induced cardiac damage [15, 16]. Based on data obtained from the use of dexrazoxane derivatives that are lacking ion-chelating activity but still harboring Topo II inhibitory potency, it is hypothesized that their cardioprotective activity mainly rests on the inhibition of Topo IIβ isoform [17, 18]. This hypothesis is supported by the observation that genetic knockout of Top2β protects from anthracycline-induced cardiotoxicity in vivo [17].

It is still unclear which cardiac cell types are of utmost relevance for the pathophysiology of Dox-induced acute and chronic cardiotoxicity. Both cardiomyocytes, endothelial cells, fibroblasts, immune cells and cardiac progenitor cells (CPC) have been suggested to contribute to Dox-mediated heart injury [19, 20]. It appears that the contribution of cardiomyocytes versus non-cardiomyocytes for cardiac damage following anthracycline exposure depends on the time point of analysis (acute vs. chronic), concrete treatment regimen (single vs. repeated treatments) and the dose (single, cumulative) applied [21, 22]. In view of the delayed onset of cardiotoxicity under clinically relevant setting of repeated Dox treatment cycles, it is tempting to speculate that Dox-induced persisting damage to CPC leads to a further impairment of the already limited regenerative capacity of the heart. Having in mind the pathophysiological relevance of endothelial damage [19, 23–26] as well as the involvement of CPC in Dox-induced cardiotoxicity [27–29], our study aims to investigate the vulnerability of differentiating endothelial progenitor cells to Dox-induced damage.

Due to their barrier function, endothelial cells are exposed to the highest concentration of intravenously administered Dox. Accordingly, we assume that error-free regenerative mechanisms of the endothelium are of particular relevance for the maintenance of cardiac health. We hypothesize that long-term deleterious effects resulting from Dox-based anticancer therapy may involve Dox-induced persisting damage to EPC that impairs their differentiation accuracy, thus eventually promoting the differentiation of surviving EPC into dysfunctional endothelial-like cells. To scrutinize this hypothesis, murine embryonic stem cells (mESC), thereof derived endothelial progenitor cells (EC d4) and differentiated endothelial-like cells (EC d6) were used as in vitro model for comparative analyses. In this model, EC d4 cells are considered as endothelial representatives of EPC. In view of the putative relevance of DNA double-strand breaks (DSBs), which are resulting from Topo II inhibition, in Dox-triggered cardiotoxicity [18, 30–33], we additionally included pharmacological inhibitors of DSB repair by homologous recombination (HR) and DNA damage response (DDR) into our study. Thereby, we aimed to examine whether insufficient processing of DNA damage that is caused by endogenous mechanisms (e.g. metabolism) also influences the fitness of EPC and their endothelial differentiation accuracy. In more detail, cell viability, proliferation, mitochondrial homeostasis, the steady state levels of DSB and SSB as well as the activation of DDR-related mechanism, including senescence, were comparatively analyzed. Most important, the outcome of drug treatment of differentiating EC d4 with respect to prototypical endothelial functions of terminally differentiated EC d6 was investigated as well. Thereby, we aim to assess possible long-term toxic effects of drug exposure of EPC on their fitness and the functional competence of the thereof differentiated endothelial progeny. We assume that the results obtained from our mESC-based in vitro model are of fundamental toxicological significance for adults and/or pediatric patients receiving Dox-containing chemotherapy. Thus, a major aim of our study was to identify potential toxicological hazards to EPC resulting from Dox exposure with regard to developmental and/or regenerative processes in the context of Dox-based anticancer therapy. On the long term, our study is anticipated to contribute to the future development of preventive strategies that lower the risk of chronic cardiotoxicity that is attributable to Dox-based treatment regimen.

## Materials and methods

### Materials

Mouse ESC (LF2) were isolated from the mouse strain 129 J [34] and originate from A. Smith (Oxford, UK). Since these cells are an established cell line, ethics approval was not needed. Activin A, BMP4, bFGF, and VEGF165 are from PeproTech (Hamburg, Germany), GSKi from Calbiochem (Darmstadt, Germany), ALKi and entinostat (EST) from Sellek Chemicals LLC (Munich, Germany), Forskolin and Hydroxyurea (HU) from Sigma-Aldrich (Munich, Germany), doxorubicin (Dox) from Cellpharm (Bad Vilbel, Germany) and B02 was from Tocris Bioscience (Bristol, UK). Antibodies originate from the following companies: VE-cadherin from eBioscience (Frankfurt, Germany), RAD51 and ZO1 from Abcam (Cambridge, UK), H3ac and H4ac from Active Motif (Carlsbad, California, USA), p-AMPKα (Thr172), Chk1, p-Chk1 (Ser345), p-GSK3β (Ser9), ac p53 (Lys383), p-p53 (Ser15), p-p70S6K (Thr389) and 53BP1 from cell signaling (Danvers, MA, USA), γH2AX from Millipore (Billerica, MA, USA), p-KAP1 (Ser824) and p-RPA32 (Ser4/Ser8) from Bethyl Laboratories (Montgomery, AL, USA), α-Tubulin ac and β-Actin from Santa Cruz (California, USA), Fluorophore-conjugated secondary antibody Alexa Fluor^®^ 488 and 555 from Life Technologies (Carlsbad, California, USA), anti-rat Alexa Fluor^®^ 488 from Abcam (Cambridge, UK) and peroxidase-conjugated secondary antibody from Rockland (Rockland, Limerick, Pennsylvania, USA). The thymidine analogues 5-Chloro-2-deoxyuridine (CldU) and 5-Iodo-2-deoxyuridine (IdU) were obtained from Sigma (Steinheim, Germany).

### Cell culture of mESC and in vitro differentiation

Mouse embryonic stem cells (LF2) were cultivated under feeder-free conditions on 0,1% gelatine coated plates using knock-out Dulbecco’s Modified Eagle Medium (KO-DMEM) supplemented with knock-out serum replacement (15%) (Gibco, Carlsbad, CA, USA), penicillin/streptomycin (1%), glutamax (1%), β-mercaptoethanol (5 × 10 − 5 M) (Invitrogen, Carlsbad, CA, USA) and leukemia inhibitory factor (LIF) (Millipore, Billerica, MA, USA) (1000 U/ml) at 37 °C in an atmosphere containing 5% CO_2_. For endothelial differentiation a modified protocol, according to Chiang and Wong [47], was used [35]. 0.15 × 10^5^ cells per 6 well were seeded in serum-free N2b27-medium supplemented with growth factors from day 2 onwards. The differentiation process into EC-like cells was completed on day 6. This differentiation protocol was proven to result in functionally competent endothelial like cells [36]. Moreover, it enables the directed differentiation of an endothelial-like cell population purity of > 90% as concluded from flow cytometry- and immunocytochemistry-based calculation of the percentage of CD31 (PECAM1) positive cells (Supplementary Fig. 1).

### Analysis of cell viability

Drug-induced cytotoxicity was monitored by analyzing cell viability using the AlamarBlue assay, which measures the reduction of the non-fluorescent dye resazurin (Sigma [Steinheim, Germany]) to fluorescent resorufin by living (i.e. metabolically active) cells. Fluorescence was measured in quadruplicates (excitation: 535 nm, emission: 590 nm (Tecan infinite 200, Tecan, Männedorf, Switzerland)). Relative cell viability of the untreated control was set to 100%.

### Analysis of cell proliferation

S-phase activity of cells was monitored by measuring EdU incorporation using the EdU-Click 488 assay according to the manufacturer’s protocol. Briefly, cells were pulse-treated with 5-ethynyl-2′-deoxyuridine (EdU) for 3 h. Alexa Fluor 488-conjugated reaction cocktail was added (30 min, RT) and nuclei were counterstained with DAPI. The percentage of EdU-positive area/DAPI-positive area was calculated by fluorescence microscopy (Olympus BX43, 20x objective).

### Quantitative gene expression analyses (RT-qPCR)

Total RNA was purified using the RNeasy Mini Kit (Qiagen, Hilden, Germany). Reverse transcriptase (RT) reaction was performed with the HighCapacity cDNA Reverse Transcription Kit (Applied Biosystems, Darmstadt, Germany) using 900–2000 ng of RNA. Quantitative RT-qPCR analysis was performed using following conditions: (1) 95 °C–10 min; (2) 45 amplification cycles with 95 °C–15 s, 55 °C–15 s, and 72 °C–17 s; (3) 95 °C–1 min, 55 °C–1 min, 65 °C–5 s. If not stated otherwise, analyses were performed in technical triplicates using pooled samples from two independent experiments (each performed with biological duplicates) by use of a CFX96 cycler (BioRad) and the SensiMix SYBR Kit (Bioline, London, UK). The primers used for mRNA expression analyses are listed in Supplementary Table 1. To ensure the specificity of the amplification product, melting curves were recorded at the end of the run. mRNA expression levels were normalized to *β-Actin* and *Gapdh*. Relative mRNA expression in mESC or corresponding untreated controls was set to 1.0. Only changes in gene expression of ≤ 0.5- and ≥ 2-fold were considered as biologically relevant. Fold-changes in gene expression are summarized in Supplementary Table 2.

### Western blot analysis

Total protein cell extracts were obtained upon sonication in RIPA buffer (EpiShearTM Probe sonicator, Active Motif [La Hulpe, Belgium]). Protein concentrations were determined using the DC Protein Assay (BioRad [Munich, Germany]). Extracts were supplemented with Roti^®^-Load buffer (Carl Roth GmbH [Karlsruhe, Germany]) followed by heating (95 °C for 5 min). 20 µg of protein was separated by SDS-PAGE and transferred onto nitrocellulose membrane. After blocking (5% non-fat milk in TBS/0.1% Tween 20 or 5% BSA in TBS/0.1% Tween 20) (1 h, RT) membrane was incubated with primary antibodies (1:500–5000; overnight, 4 °C). After washing with TBS/0.1% Tween 20, peroxidase-conjugated secondary antibody (1:2000) was added (2 h, RT) and bands were visualized using ChemiDoxTM Touch imaging system (BioRad, Munich, Germany). Quantitative data obtained from densitometrical analysis are summarized in Supplementary Table 3.

### Immunofluorescence analysis of tight junctions

The expression of the intercellular tight junction protein ZO1 was analyzed by immunocytochemistry. After fixation (3,7% formaldehyde/PBS; 15 min, RT), permeabilization (0.1% TritonX/PBS, 10 min, RT) and blockage (5% BSA/PBS; 1 h, RT), cells were incubated with primary antibody against ZO1 (1:100; 4 °C, overnight). After washing with PBS fluorescence-labeled secondary antibody (Alexa Fluor 488 goat polyclonal to rabbit or rat) was added (1:1000; 2 h, RT). Nuclei were counterstained with DAPI before microscopic analysis was performed (Olympus BX43 fluorescence microscopy; 100x objective).

### Analysis of DNA damage formation and DNA repair

DNA double-strand breaks were monitored by immunocytochemical detection of nuclear γH2AX and 53BP1 foci [37, 38]. Cells were fixed with 3.7% formaldehyde/PBS (15 min, RT) and permeabilized (0.3% TritonX/PBS; 15 min, RT). After blocking (5% BSA in 0.3% TritonX/PBS (1 h, RT)) incubation with γH2AX antibody (1:1000) and 53BP1 antibody (1:500) was done (overnight, 4 °C) followed by incubation with fluorescence-labeled secondary antibody (1:500, 2 h, RT). Nuclei were counterstained with DAPI and analyzed by fluorescence microscopy (Olympus BX43, 100x objective). In addition, DNA strand breaks and apurinic/apyrimidinic sites were analyzed using the alkaline comet assay [39]. Cell suspension was mixed with 0.5% low melting point agarose and applied onto glass slides coated with 1.5% agarose. After lysis in alkaline buffer (pH 10, 1 h, 4 °C, protected from light) and DNA denaturation in precooled electrophoresis buffer (pH > 13, 25 min, 4 °C, protected from light) electrophoresis was performed (300 mA, 25 V, 4 °C, 25 min). After neuralization, DNA was stained with propidium iodide (50 µg/mL). Comets were analyzed via fluorescence microscopy using TriTek Comet ScoreTM software (version 1.5), evaluating 50 cells per experimental condition.

### Analysis of doxorubicin transport

Import and export of Dox were examined via flow cytometry. Cells were pulse-treated with 0.25 µM and 1 µM Dox for 2 h (import) followed by a 6 h post-incubation period with fresh medium (export). Trypsinized cells were washed with PBS and pelleted by centrifugation (200 x g, 5 min, 4 °C). After additional washing, cells were resuspended in PBS. Quantification of Dox import/export was done by flow cytometry-based analyses (excitation: 488 nm, filter: 585/40; Becton Dickinson, AccuriTM C6 plus (Heidelberg, Germany)). Dox import and export are reflected by the increase and decrease, respectively, in mean fluorescence intensity.

### DNA fiber spreading assay

To analyze DNA replication fork progression, the DNA fiber spreading assay was performed [40, 41]. Briefly, cells were pulse-labeled with 20 µM CldU (20 min, 37 °C, 5% C0_2_) and immediately afterwards with 200 µM IdU (20 min, 37 °C, 5% C02). Cell suspension was applied onto glass slides followed by cell lysis (0.5% SDS, 200 mM Tris–HCl, 50 mM EDTA). After incubation (6 min, RT), slides were tilted upwards to stretch the fibers, dried 6 min lying horizontally flat and fixed (5 min, RT) in methanol: acetic acid (3:1). After drying (7 min, lying horizontally flat) slides were stored overnight in 70% ethanol (4 °C). The next day samples were incubated in 100% methanol (5 min, RT) followed by denaturation in 2.5 M HCl (1 h, RT) and blocking (5% BSA in PBS, 1 h, 37 °C). DNA Fibers were stained with rat anti-BrdU (1:40) for CldU detection and mouse anti-BrdU (1:70) for IdU detection (in 0.5% BSA/PBS; 1 h, RT). After washing (0.05% Tween 20 in PBS and PBS) incubation with secondary antibodies anti-rat 488 (1:400) and anti-mouse 555 (1:250) was performed (1 h, RT, 0.5% BSA in PBS). Following washing with 0.05% Tween 20 in PBS and PBS, the slides were mounted with Fluoroshield (Sigma [Steinheim, Germany]). DNA fibers were analyzed microscopically (Olympus BX43 fluorescence microscope [40 objective]). The length of CldU (green) and IdU (red) tract was measured using ImageJ software, analyzing 200 fibers per experimental condition.

### Mitochondrial analysis - mitostaining

Mitochondrial membrane potential (ΔΨ_m_) was analyzed by live-cell imaging using TMRM-MitoTracker Green co-staining. Cells were seeded and differentiated on Poly-D-Lysine-fibronectine-coated live-imaging dishes. For staining, cells were incubated with 200nM MitoTracker Green (Invitrogen) and 50 nM TMRM (Invitrogen) in DMEM (30 min, 37 °C, 5% CO_2_). After washing with PBS, live-cell microscopy was performed using a spinning disc confocal microscope (PerkinElmer) equipped with a 60x oil-immersion objective (N.A = 1.49) and a Hamamatsu C9100 camera (1000 × 1000 pixel) (excitation (MitoTracker Green): 488 nm, excitation (TMRM): 561 nm). The cells were kept at 37 °C in OptiMEM supplemented with 10 mM HEPES during imaging. The images were obtained at emission wavelength of 527 nm (W55) and 615 nm (W70) for 488 nm and 561 nm excitation, respectively. The mean intensity of TMRM was calculated using ImageJ software, analyzing 50 mitochondria per experimental condition (EC d4 + Dox 0.05, EC d6 + Dox 0.1 µM: ≥ 20 mitochondria).

### Functional analysis of EC

To monitor prototypical functions of endothelial cells, the uptake of fluorescent 1,1-dioctadecyl-3,3,3,3-tetra-methylindocarbocyanine-labeled acetylated LDL (Dil-acLDL) was examined. Briefly, cells were incubated with Dil-acLDL (10 µg/ml, 4 h, 37 °C). After fixation (3.7% cold formaldehyde/PBS (15 min, RT) nuclei were counterstained with DAPI. The uptake of DiI-acLDL was analyzed by fluorescence microscopy (Olympus BX43, 20x objective). Additionally, the response of differentiated EC to inflammatory cytokines was measured. After treatment with a pro-inflammatory cytokine mixture (TNFα/IL-1β, 10 ng/mL) for 1 h, mRNA expression of activated EC markers *E-selectin*,* Icam-1*,* Vcam-1*,* Ccl2*,* eNos*,* iNos* was analyzed (RT-qPCR).

### Analysis of senescence - beta-galactosidase staining

Beta-galactosidase activity was analyzed using the Cellular Senescence Assay Kit (Cell Biolabs, Inc., San Diego, CA, USA) according to the manufacturer’s instructions. After treatment and/or post-incubation period, the cells were fixed and stained (overnight, 37 °C). For control, untreated cells were incubated with working solution containing citrate-Na_2_HPO_4_ buffer at pH 4. The cells were analyzed by light microscopy (Axiovert 40 C, Carl Zeiss AG, 20x objective) and the fraction of beta-galactosidase positive (blue) cells was calculated ($\:\ge\:$5 images per sample).

### Statistical analysis

For statistical analysis, the two-tailed unpaired Student’s t-test and One-way ANOVA with Tukey´s and Dunnett´s post-hoc test were employed (using GraphPad Prism 10 software) as mentioned in the legends. P-values ≤ 0.05 were considered as statistically significant.

## Results

### Drug treatment influences the viability of mESC, EC d4 and EC d6 as well as the differentiation potential of ECd4 in an agent and cell-type specific manner

**Fig. 1:**
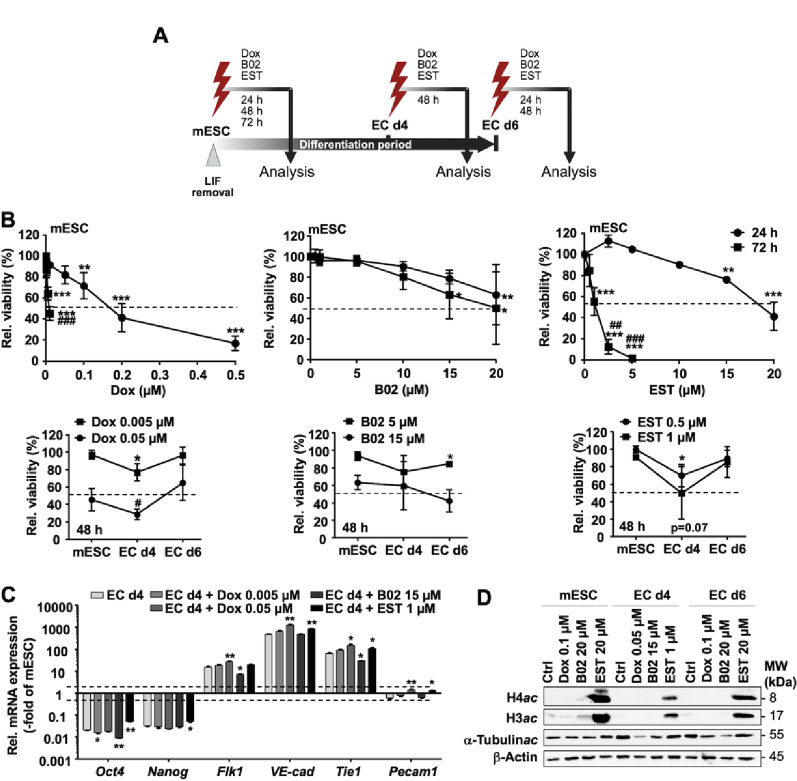
Endothelial progenitor cells (EC d4) reveal high sensitivity to Dox and epigenetic modulator. **A**: Schematic overview of the treatment scheme in course of the differentiation of mESC into EC d6. mESC were treated 24 h after seeding with Dox, B02 or EST for a time period of 24–72 h before analyses were performed. Differentiation was induced by removing LIF and adding serum-free N2b27 medium. On day 2 and day 4 of the differentiation process, medium was supplemented with growth factors and small molecules required for endothelial lineage differentiation. To analyze drug-induced stress responses, cells were either treated in an endothelial progenitor state on day 4 (EC d4) for 48 h (i.e., during ongoing differentiation) or after terminal differentiation on day 6 (EC d6) for 24–48 h without growth factor supplement before analyses were performed (created with BioRender.com). **B**: Viability of mESC, EC d4 and EC d6 was measured after Dox, B02 and EST treatment using the AlamarBlue assay as described in the methods. Upper panel: mESC were treated for 24–72 h with the compounds. Lower panel: Cells were treated for 48 h (EC on day 4 and 6 continuing the differentiation protocol, measured on day 6 and 8). Data represent the mean ± SD of three independent experiments performed in quadruplicates. The dashed lines show the IC_50_. One-way ANOVA: *, *p* ≤ 0.05; **, *p* ≤ 0.01; ***, *p* ≤ 0.001 as compared to untreated control. Student‘s t–test: ^##^, *p* ≤ 0.01; ^###^, *p* ≤ 0.001 drug treatment 72 h vs. 24 h treatment. **C**: RT-qPCR gene expression analysis of prototypical stem cell (*Nanog*,* Oct4*), mesodermal progenitor cell (*Flk1*) and endothelial cell marker genes (*VE-cad*,* Pecam1*,* Tie1*). Cells were treated with Dox, B02 and EST on day 4 for 48 h continuing the differentiation protocol. mRNA expression was analyzed on day 6 (terminally differentiated EC). Relative mRNA expression of mESC was set to 1.0. Data represent the mean ± SEM of two independent experiments with two pooled samples per condition, each performed in triplicates. One-way ANOVA (Dox), Students t-test (B02, EST): *, *p* ≤ 0.05; **, *p* ≤ 0.01 as compared to untreated control. **D**: To monitor drug-induced effects on differentiation-associated epigenetic mechanisms related to histone acetylation, the protein expression of acetylated Histone3 (H3*ac*) and 4 (H4*ac*) as well as acetylated α-Tubulin (α-Tubulin*ac*) was analyzed in mESC, EC d4 and EC d6 after Dox, B02 or EST treatment (mESC, EC d6: 24 h; EC d4: 48 h). Protein expression of β-Actin was used as loading control. See also Supplementary Table 3

To comparatively analyze the Dox response of mESC, differentiating EPC (EC d4) and differentiated (EC d6) endothelial-like progeny, cell viability was analyzed up to 72 h after drug addition (Fig. 1A). For control, pharmacological inhibitors of DSB repair and DDR-related factors were included. Here, B02 which inhibits the HR-related DSB repair protein RAD51 [42, 43] and the HDAC class I inhibitor entinostat (EST) that interferes with mechanisms of DSB repair and DDR [44, 45], were employed. Treatment of mESC with Dox for 72 h reduces cell viability much stronger than a 24 h treatment period as concluded from the IC_50_ values obtained from AlamarBlue-based cytotoxicity analyses (IC_50_ 24 h: 0.17 µM; IC_50_ 72 h: 0.009 µM) (Fig. 1B). Identical effects were observed following EST exposure (IC_50_ 24 h: 18.8 µM; IC_50_ 72 h: 2.6 µM). By contrast, cytotoxicity evoked by B02 was very similar after a 24–72 h treatment period (IC_50_ ∼20 µM) (Fig. 1B). To address the question whether drug-induced cytotoxicity changes during differentiation, mESC, EC d4 and EC d6 were exposed to Dox or pharmacological inhibitors and cell viability was comparatively analyzed 48 h later (Fig. 1B). The data obtained demonstrate a significant higher Dox and EST susceptibility of EC d4 as compared to both mESC and EC d6 (Fig. 1B), pointing to a transient particularly drug sensitive time window during differentiation. Notably, this difference was not observed for the RAD51 inhibitor B02 (Fig. 1B). Under our experimental setting of permanent drug treatment, the drug sensitivity of mESC and EC d6 was similar (Fig. 1B).

In view of the transient Dox and EST hypersensitivity of differentiating EC d4, we investigated whether drug treatment of EC d4 impacts their further differentiation into EC d6. To this end, EC d4 were treated during the remaining differentiation period (i.e., 48 h) with Dox, EST or B02 and the mRNA expression of prototypical stem cell factors (*Oct4*,* Nanog*) and endothelial factors (*Flk1*,* VE-cadherin*,* Tie1*,* Pecam1*) was analyzed in differentiated EC d6 (Fig. 1C). We found moderate, yet statistically significant, differences between the various experimental groups. Dox treatment of EC d4 did not impact the further decrease in the mRNA expression of stem cell-related factors in differentiated EC d6, indicating that Dox-induced damage in EC d4 does not influence their differentiation dependent loss of pluripotency. Importantly yet, Dox exposure of EC d4 resulted in an upregulated mRNA expression of the EC-related marker genes *Flk1*,* VE-cadherin*,* Tie1* and *Pecam1* in differentiated EC d6 (Fig. 1C), indicating that Dox promotes the differentiation of endothelial progenitors. The outcome of B02 and EST treatment was different from that of Dox exposure. EST treatment of EC d4 mitigated the differentiation-dependent decrease in *Oct4* and *Nanog* mRNA expression and increased the mRNA expression of *VE-cadherin* and *Tie1* in terminally differentiated EC d6. By contrast, treatment of EC d4 with B02 promoted the differentiation dependent reduction in *Oct4* mRNA expression while inhibiting the mRNA expression of *Flk1* and *Tie1* in EC d6 (Fig. 1C). In view of the complex epigenetic alterations going along with differentiation, we exemplarily monitored the acetylation status of nuclear histone proteins (H3, H4) and cytosolic α-Tubulin by western blot under basal situation and following drug exposure. Basal protein levels of H3ac and H4ac are very low in both mESC, EC d4 and EC d6 and specifically increased in response to EST treatment only (Fig. 1D). Notably, this EST-triggered response was most profound in mESC and weakest in EC d4 cells. The acetylation level of α-tubulin was moderately reduced in all cell types under investigation upon treatment with Dox and B02 but not EST (Fig. 1D).

### Differentiation dependent alterations in drug transport

**Fig. 2:**
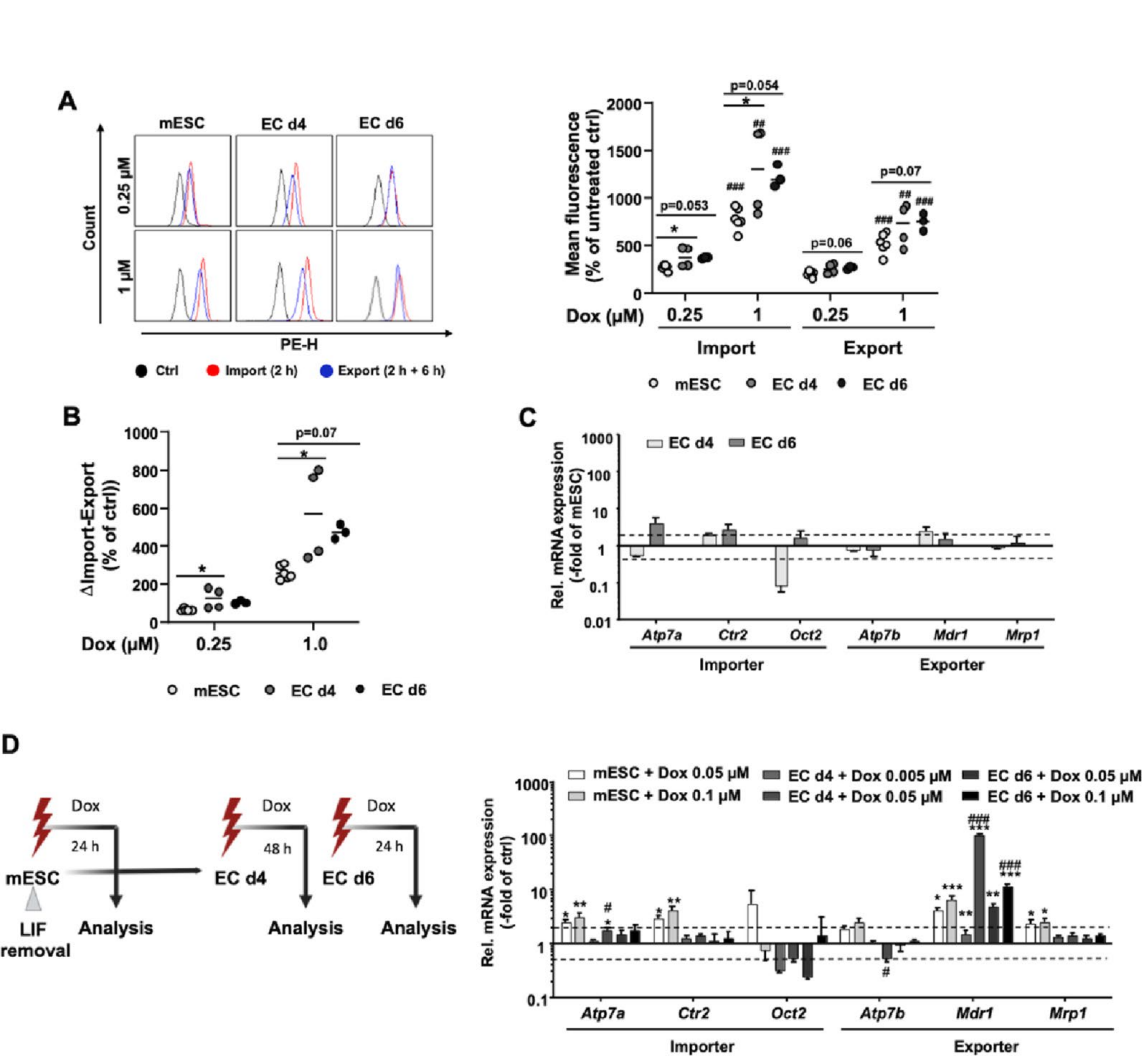
Differentiation dependent alterations in drug transport. **A**: Intracellular doxorubicin (Dox) fluorescence was measured via flow cytometry. mESC, EC d4 and EC d6 were treated for 2 h with 0.25 µM and 1 µM Dox. To analyze Dox import, the increase in mean fluorescence intensity was measured immediately after pulse treatment or after a 6 h post incubation period (export). One-way ANOVA: *, *p* ≤ 0.05; **, *p* ≤ 0.01 as compared to mESC. Student‘s t–test: ^##^, *p* ≤ 0.01; ^###^, *p* ≤ 0.001 (1 µM vs. 0.25 µM). **B**: To calculate the export capacity the Dox efflux normalized to the corresponding untreated control was calculated. Data represent three to four independent experiments. One-way ANOVA: *, *p* ≤ 0.05* as compared to mESC. **C**: Basal mRNA expression of selected drug importer (*Atp7a*,* Ctr2*,* Oct2*) and exporter (*Atp7b*,* Mdr1*,* Mrp1*) genes of EC d4 and EC d6 in relation to mESC. Data show the mean ± SEM of two to three independent experiments with two pooled samples, each performed in duplicates. Changes in mRNA levels of ≥ 2.0 and ≤ 0.5 indicated by dashed lines are considered as biologically relevant. See Supplementary Fig. 2 for the analysis of basal protein expression of the transporters. **D**: Schematic overview of the drug-treatment scheme (left panel; created with BioRender.com). Right panel: Comparative RT qPCR analysis of mRNA expression of selected drug transporter genes of mESC, EC d4 and EC d6 after Dox exposure (mESC, EC d6: 24 h; EC d4: 48 h) Relative mRNA expression of the corresponding untreated control was set to 1.0. Data show the mean ± SEM of two to three independent experiments with two pooled samples per condition, each performed in duplicates. Changes in mRNA levels of ≥ 2.0 and ≤ 0.5 indicated by dashed lines are considered as biologically relevant. One-way ANOVA: *, *p* < 0.05; **, *p* < 0.01; ***, *p* < 0.001 as compared to mESC. ^#^, *p* < 0.05; ^###^, *p* < 0.001 as compared to lower drug concentration

Since Dox is subject of active transport mechanisms, we investigated whether mESC, EC d4 and EC d6 differ in their Dox import and export activity by exploiting the inherent reddish fluorescence of doxorubicin. Measuring drug uptake after a 2 h Dox pulse-treatment period (0.25 µM, 1.0 µM), we found a concentration- and differentiation status-dependent increase in intracellular Dox levels. At low Dox concentration (i.e. 0.25 µM), a significantly increased drug import was only detected in EC d4 as compared to mESC (Fig. 2A). Using higher Dox concentration (1 µM), further significant increase in drug import was found, with EC d4 and EC d6 revealing the highest fluorescence (Fig. 2A). To monitor drug export, residual intracellular Dox fluorescence was measured after a 6 h post-incubation period in the absence of Dox. Drug export kinetics depended on the Dox concentration used for the initial pulse-treatment (Fig. 2A). The residual intracellular Dox level was tendentially higher in EC d6 than mESC. Calculation of the Dox efflux capacity revealed an enhanced export activity of EC d4 as compared to the other differentiation stages (Fig. 2B). Analyzing the mRNA expression of individual drug importers and exporters under basal situation we found noticeable (i.e. <0.5>2.0-fold) differences in the expression of importers *Atp7a*,* Ctr2* and *Oct2* and the exporter *Mdr1* between mESC and both EC d4 and EC d6 (Fig. 2C). Of note, differences in basal mRNA expression levels of these transporters were not reflected on the protein level (Supplementary Fig. 2), emphasizing the need for measuring Dox transport activity for meaningful conclusions. Following Dox treatment for 24 h, the mRNA expression of the drug importers *Atp7a*, *Ctr2* and *Oct2* was specifically enhanced in mESC (Fig. 2D). Among the drug exporters, the most substantial Dox-stimulated increase was observed for *Mdr1*, with EC d4 showing the strongest response at an equimolar Dox concentration of 0.05 µM (Fig. 2D). This finding indicates that the observed elevated Dox export activity of EC d4 (see Fig. 2B) is due to an enhanced MDR1-related drug transport [46], though the contribution of other drug exporters can´t be excluded.

### Differentiation dependent formation of DNA damage by doxorubicin

**Fig. 3:**
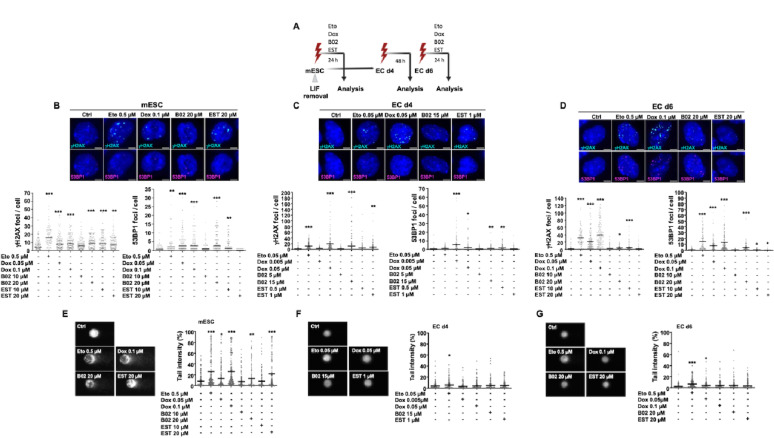
The type of steady state DNA damage is agent- and cell type-specific. Cells were treated undifferentiated (mESC) and terminally differentiated (EC d6) on day 6 for 24 h or on day 4 (EC d4) for 48 h continuing differentiation protocol with 1–2 equitoxic doses of Dox, EST and B02. As an additional control, cells were treated with one concentration of the Topo II inhibitor etoposide (Eto). **A**: Schematic overview of the drug-treatment scheme in the course of the differentiation process (created with BioRender.com). **B**–**D**: Results of immunocytochemical analyses of the steady-state number of nuclear γH2AX (cyan) and nuclear 53BP1 (magenta) foci per cell (Dox, B02, EST: *n* = 3, *N* = 50; Eto: *n* = 2–3, *N* = 50) in mESC (**B**) EC d4 (**C**) and EC d6 (**D**) following drug treatment. One-way ANOVA (Dox, B02, EST); t-test (Eto); *, *p* ≤ 0.05; **, *p* ≤ 0.01; ***, *p* ≤ 0.001 as compared to the corresponding untreated control. Scale bar: 5 μm **E**–**G**: The steady state levels of DNA single-strand breaks (SSB), DNA double-strand breaks (DSB) in mESC (**E**), EC d4 (**F**) and EC d6 (**G**) after treatment was analyzed by use of the alkaline comet assay. Quantification of DNA in tail (%) was performed as described in the methods (*n* = 3, *N* = 50). (**E**) One-way ANOVA (Dox, B02, EST); Student’s t-test (Eto); (**F**,** G**) One-way ANOVA (Dox); Student’s t-test (Eto, B02, EST); *, *p* ≤ 0.05; **, *p* ≤ 0.01; ***, *p* ≤ 0.001 as compared to the corresponding untreated control.

Since Dox causes the formation of highly cytotoxic DSB by inhibition of Topo II, the steady state level of DSB was comparatively investigated in mESC, EC d4 and EC d6 by monitoring the number of nuclear γH2AX and 53BP1 foci, which are well accepted surrogate markers of DSB [37, 47–49] (Fig. 3B-D). For these analyses, equitoxic drug concentrations were used for treatment (Fig. 3A). A significant increase in nuclear γH2AX foci was observed in mESC following treatment with Dox and the Topo II inhibitor etoposide (Eto), which was included for control, as well as following B02 and EST exposure (Fig. 3B). Similar results were obtained measuring nuclear 53BP1 foci (Fig. 3B), which are considered as marker of DSB that are processed by NHEJ [38, 50]. Similarly, EC d4 also responded to treatment with Dox, Eto, B02 or EST with a clear increase in DSB numbers (Fig. 3C). As opposed to mESC, EC d6 revealed a significant increase DSB formation upon Eto and Dox exposure and, to a minor extent, following treatment with the DDR/repair inhibitors (Fig. 3D). Moreover, the steady-state number of Dox-induced γH2AX and 53BP1 foci was highest in EC d6 (Fig. 3B-D). Measuring the steady-state level of SSB by use of the alkaline comet assay, a significant increase in SSB was observed in mESC treated with Eto, Dox or DDR modulators (Fig. 3E), while no or only a minor increase in SSB occurred in EC d4 and EC d6 (Fig. 3F and G). In order to specifically assess the DSB repair capacity of cells at different stages of differentiation, the cells were exposed to a single dose of ionizing radiation (IR), since IR-induced DNA damage stems directly from physical interactions with DNA. Subsequently, the temporal decline of IR-induced DSBs was examined as a surrogate marker of DSB repair activity by monitoring the number of nuclear γH2AX foci (general DSB marker) as well as of 53BP1 foci (marker of NHEJ-associated DSB repair). The results of these molecular analyses revealed a similar IR-induced increase in DSB numbers as measured 30 min after irradiation. We did not observe differentiation-related differences in total DSB repair capacity, as reflected by the number of residual γH2AX foci found after a post-incubation period of 4 h (Supplementary Fig. 3A, 3B). However, a higher number of residual large-sized 53BP1 foci was observed after post-incubation period of 4 h in EC d4 and EC d6, indicating a differentiation-dependent shift in the choice of the preferred DSB repair pathway from HR towards error-prone NHEJ (Supplementary Fig. 3C).

### Dox and pharmacological inhibitors differently impact replicative stress responses of mESC, EC d4 and EC d6

**Fig. 4:**
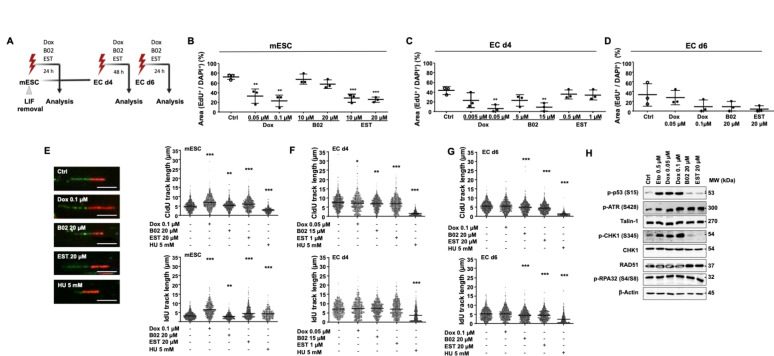
Influence of Dox, B02 and EST on proliferation and activation of replication stress-associated mechanisms. Cells were treated undifferentiated and terminally differentiated on day 6 for 24 h or on day 4 for 48 h continuing the differentiation protocol after drug treatment with the indicated concentrations of Dox, EST or B02. **A**: Schematic illustration of the treatment scheme (created with BioRender.com). **B**–**D**: Cell proliferation was measured after drug treatment by monitoring EdU incorporation (3 h EdU pulse-treatment) The percentage of EdU^+^ cells was calculated by analyzing the EdU^+^ area in correlation to the nuclear DAPI counterstain (fluorescence microscopy). Data represent the mean ± SD of three independent experiments. **B**,** C** One-way ANOVA; **D** One-way ANOVA (Dox), Student’s t-test (B02, EST), *, *p* ≤ 0.05; **, *p* ≤ 0.01; ***, *p* ≤ 0.001 as compared to the corresponding untreated control. **E**–**G**: Replication fork progression was analyzed by use of the DNA fiber spreading assay as described in methods. Data shown were obtained from *n* = 3–4 independent experiments with 200 fibers being analyzed per experimental sample. For additional control, cells were treated with the ribonucleotide reductase inhibitor hydroxy-urea (HU; 5 mM, 1 h) (*n* = 2, *N* = 1–2). Student´s t-test: *, *p* ≤ 0.05; **, *p* ≤ 0.01; ***, *p* ≤ 0.001 as compared to the corresponding untreated control. **E**(left panel): Representative images of immunocytochemical analyses of CldU (green)/IdU (red) containing DNA fibers in mESC (Scale bar 5 μm). **H**: Protein expression of selected replication stress response-related factors of non-treated mESC and following 24 h of Eto, Dox, B02 and EST treatment were analyzed by Western blot. Protein expression of β-Actin and Talin-1 were used as loading controls. See also Supplementary Table 3

To monitor alterations in proliferation going along with differentiation and/or drug treatment, S-phase activity of mESC, EC d4 and EC d6 was monitored by measuring the incorporation of EdU. Under basal situation, the percentage of EdU-positive cells dropped with the differentiation status from 70% EdU-positive mESC to 45 − 30% EdU-positive EC d4 and EC d6 (Fig. 4B-D). Having in mind the differentiation stage-dependent variations in basal proliferation activity, both equimolar and equitoxic drug concentrations were employed when analyzing their impact on S-phase activity. Dox treatment caused a significant drop in the number of EdU-positive cells in mESC (Fig. 4B). Similar effect was found upon treatment of mESC with EST but not B02 (Fig. 4B). By contrast, EC d4 revealed a decrease in S-phase activity following Dox and B02 treatment but not after EST exposure (Fig. 4C). Noteworthy, the number of EdU-positive EC d6 cells remained unaffected by drug treatment, even at the highest Dox concentration (0.1 µM) used. To further clarify the impact of the drugs on S-phase activity, replication fork progression was investigated using the DNA fiber spreading assay. Untreated mESC displayed a reduced replication fork progression as compared to EC d4 (Fig. 4E-F). The ribonucleotide reductase inhibitor hydroxy-urea (HU) decreased replication fork speed in all cell-types under investigation, with EC d4 revealing the strongest response (Fig. 4E-G). Inhibition of RAD51, which is involved in the regulation of HR and of replicative stress responses [51, 52] slowed down replication fork speed in mESC, EC d4 and EC d6 (Fig. 4E-G). By contrast, interference with DDR and/or DNA repair by the class I HDACi EST [53] promoted new DNA strand synthesis in mESC, while having the opposite effects in both EC d4 and EC d6 (Fig. 4E-G). Exposure of mESC to Dox also accelerated fork progression but with visible gaps within the track (Fig. 4E). By contrast, Dox did not influence fork speed in EC d6 (Fig. 4F-G).

In extension to this data, the activation status of DDR-associated factors was monitored by Western blot analysis. Both Dox and Eto exposure caused an increase in the protein level of Ser15-phosphorylated p53 in mESC, while B02 and EST were ineffective (Fig. 4H). Having in mind the key regulatory function of ATR in the regulation of replication-associated stress responses [54], the protein level of p-ATR, the ATR-substrate CHK1 (p-CHK1) and the ssDNA binding proteins RPA (p-RPA32) and RAD51 were monitored. Dox treatment increased the level of p-ATR and p-Chk1 in mESC (Fig. 4H), supporting the hypothesis of ongoing replicative stress. Noteworthy, B02 and EST also increased the protein level of p-ATR but not of p-Chk1 (Fig. 4H). None of the drug treatments substantially affected the levels of p-RPA32 or RAD51 (Fig. 4H).

### Differentiation dependent activation of mechanisms of the DDR by Dox and DDR / DNA repair inhibitors

**Fig. 5:**
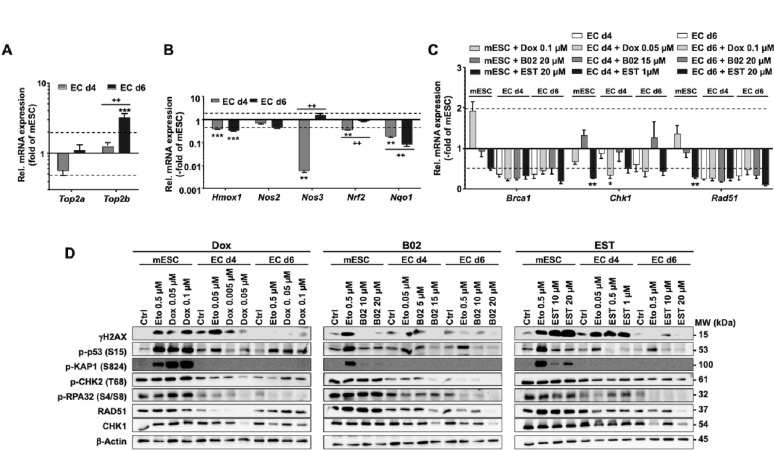
Differentiation dependent activation of mechanisms of the DDR by Dox and DDR/DNA repair inhibitors. **A**: RT-qPCR analysis of basal mRNA expression of *Top2a* and *Top2b* in EC d4 and EC d6 as compared to mESC. Data show the mean ± SEM of three independent experiments each performed in biological duplicates. Changes in mRNA levels of ≥ 2.0 and ≤ 0.5 are indicated by dashed lines and are considered as biologically relevant. One-way ANOVA: ***, *p* ≤ 0.001 as compared to mESC; ^++^, *p* ≤ 0.01 EC d4 vs. EC d6. **B**: RT-qPCR analysis of the basal mRNA expression of antioxidative response-related factors in EC d4 and EC d6 as compared to mESC. Data show the mean ± SEM of three independent experiments with two pooled samples, each performed in duplicates. Changes in mRNA levels of ≥ 2.0 and ≤ 0.5 are indicated by dashed lines and are considered as biologically relevant. One-way ANOVA: *p* ≤ 0.01** *p* ≤ 0.001*** as compared to mESC; ^++^, *p* ≤ 0.01 EC d4 vs. EC d6. **C**: Comparative analysis of mRNA expression of HR- and DDR-associated factors *Brca1*,* Rad51* and *Chk1*, respectively, following Dox, B02 and EST treatment of mESC, EC d4 and EC d6 (mESC, EC d6: 24 h; EC d4: 48 h). mESC and terminally differentiated EC d6 were treated for 24 h, while EC d4 were exposed during the whole differentiation period (i.e. 48 h). Data represent the mean ± SEM of two independent experiments each performed in biological duplicates. Changes in mRNA levels of ≥ 2.0 and ≤ 0.5 are indicated by dashed lines and are considered as biologically relevant. Relative mRNA expression of mESC was set to 1.0. Student´s t-test: *, *p* ≤ 0.05; ** *p* ≤ 0.01 as compared to the corresponding untreated control. **D**: Western Blot-based analysis of the protein expression of selected DNA repair and DDR-related factors. mESC and EC d6 were treated 24 h and EC d4 for 48 h with each two concentrations of Dox, B02 or EST as indicated. As additional control, cells were treated with etoposide (Eto). The protein expression of β-Actin was analyzed as loading control. See also Supplementary Table 3

To figure out whether the expression of Dox target proteins changes during differentiation, we comparatively analyzed the mRNA expression of the Topo II isoforms in mESC, EC d4 and EC d6. The mRNA expression of *Top2a* remained unaffected during differentiation, while we observed an upregulation of *Top2b* mRNA levels in EC d6 as compared to mESC and EC d4 (Fig. 5A). Since Dox can also evoke oxidative stress, the mRNA expression of antioxidative factors was monitored. These analyses revealed a downregulation of *Hmox1* and *Nqo1* mRNA expression in EC d4 and EC d6 as compared to mESC (Fig. 5B). In addition, a selective downregulation of *Nrf2* and *Nos3* mRNA expression was found in EC d4 but not in EC d6 (Fig. 5B), showing that transient changes in the expression of antioxidative functions occur during endothelial differentiation of mESC.

Since Dox causes the formation of DSB that are subject of DSB repair involving HR and both B02 and EST are known to impair DSB repair and DDR-related factors like RAD51, BRCA1 and CHK1 [44, 55–57], we monitored differentiation dependent alterations in the mRNA (Fig. 5C) and protein expression (Fig. 5D) of these factors. The mRNA expression of *Brca1*, *Chk1*, *Rad51* remained largely unaffected or was decreased both under basal situation and following drug treatment in all stages of differentiation (Fig. 5C). EST downregulated the mRNA expression of *Chk1* and *Rad51* in mESC while having no effect in the other cell types (Fig. 5C). Western blot-based analysis of DDR-related proteins revealed a strong increase in Serine139-phosphorylated histone H2AX (γH2AX) in mESC treated with the Topo II poisons Dox and Eto or the HDAC_i_ EST, while the Rad51_i_ B02 had no effect (Fig. 5D, middle panel). Drug-induced increase in p-p53 levels was only detectable after Dox treatment of mESC and EC d6 but not of EC d4 (Fig. 5D, left panel), indicating that specifically Dox-stimulated activation of p53-related signaling is compromised in differentiating EPC. In addition, a substantial Dox-induced increase in the protein level of Ser824-phosphorylated chromatin regulatory factor Kap1 was observed in mESC only but not in EC d4 and differentiated EC d6 (Fig. 5D). Similar effects were observed following Eto treatment (Fig. 5D). Neither EST nor B02 simulated an increase in p-KAP1 protein expression in EC d4 and EC d6, while EST was effective in mESC only (Fig. 5D). The data indicate that drug treatment may interfere with differentiation-associated epigenetic alterations in an agent- and differentiation-stage dependent manner.

### Drug treatment of progenitor EC d4 influences mitochondrial membrane potential of differentiated EC d6

**Fig. 6:**
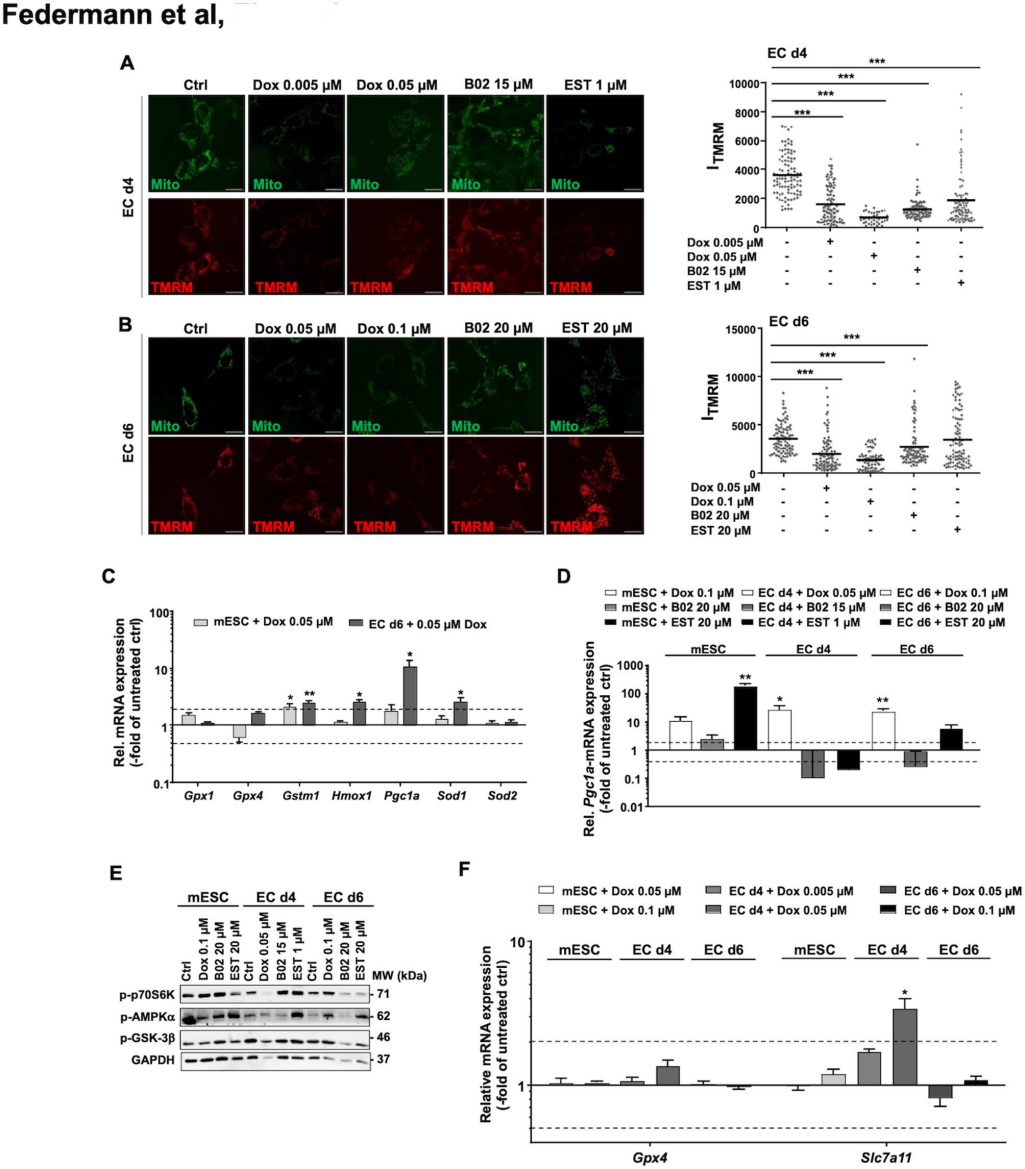
Drug treatment of progenitor EC d4 influences mitochondrial membrane potential of differentiated EC d6. Mitochondrial membrane potential (ΔΨ_m_) after drug treatment was analyzed via a live-cell imaging of a TMRM-MitoTracker^™^ Green co-staining. **A**,** B**: Cells were treated on day 4 of the differentiation process (EC d4) for 48 h with Dox, B02 or EST and mitochondrial potential was analyzed in differentiated EC d6 (**A**). For control, terminally differentiated EC d6 were treated for 24 h with Dox, B02 or EST (**B**). Following drug treatment, cells were incubated for 30 min with MitoTracker^™^ Green and TMRM. Green color show mitochondria and red indicates an intact membrane potential. Membrane integrity (fluorescence intensity) was determined as described in methods. Data show the mean of two independent experiments (*N* = 50, EC d4 + Dox 0.05 µM/ EC d6 + Dox 0.1 µM: *N* ≥ 20). One-way ANOVA (Dox); t-test (B02, EST); *, *p* ≤ 0.05; ***, *p* ≤ 0.001; as compared to the respective untreated control. Scale bar 20 μm. **C**: Relative mRNA expression of a selected subset of mitochondria-related genes was analyzed in mESC and EC d6 after 24 h Dox exposure (0.05 µM) by RT-qPCR. Relative mRNA expression of genes in the corresponding untreated control was set to 1.0. Data represent the mean ± SEM of triplicate determinations. Only changes in mRNA levels of ≥ 2.0 and ≤ 0.5 were considered as biological relevant (dashed lines). Student’s t-test: *, *p* ≤ 0.05; **, *p* ≤ 0.01 as compared to the respective untreated control. **D**: Comparative RT-qPCR gene expression analysis of *Pgc1* mRNA expression in mESC, EC d4 and EC d6 after treatment with Dox, B02 and EST (mESC, EC d6: 24 h; EC d4: 48 h). Relative mRNA expression of the corresponding controls was set to 1.0. Data represent the mean ± SEM of two independent experiments with two pooled samples, each performed in duplicates. Changes in mRNA levels of ≥ 2.0 and ≤ 0.5 indicated by dashed lines are considered as biologically relevant. Student’s t-test: **, *p* ≤ 0.01; ***, *p* ≤ 0.001 as compared to the respective untreated control. **E**: Western Blot analysis of activated (i.e. phosphorylated) protein kinases involved in the regulation of metabolism (p-p70S6K, p-AMPKα and p-GSK-3β) in mESC, EC d4 and EC d6 following Dox, B02 and EST treatment as described above. β-Actin protein expression was used for protein loading control. See also Supplementary Table 3. **F**: Relative mRNA expression of a selected ferroptosis-related marker genes was analyzed in mESC, EC d6 (24 h) and EC d4 (48 h) by RT-qPCR. Relative mRNA expression of genes in the corresponding untreated control was set to 1.0. Data represent the mean ± SEM from duplicate determinations using pooled samples obtained from *n* = 2–3 independent experiments with biological duplicates. Only changes in mRNA levels of ≥ 2.0 and ≤ 0.5 were considered as biological relevant (dashed lines). One-way ANOVA: *, *p* ≤ 0.05; **, *p* ≤ 0.01 as compared to the respective untreated control

**Fig. 7:**
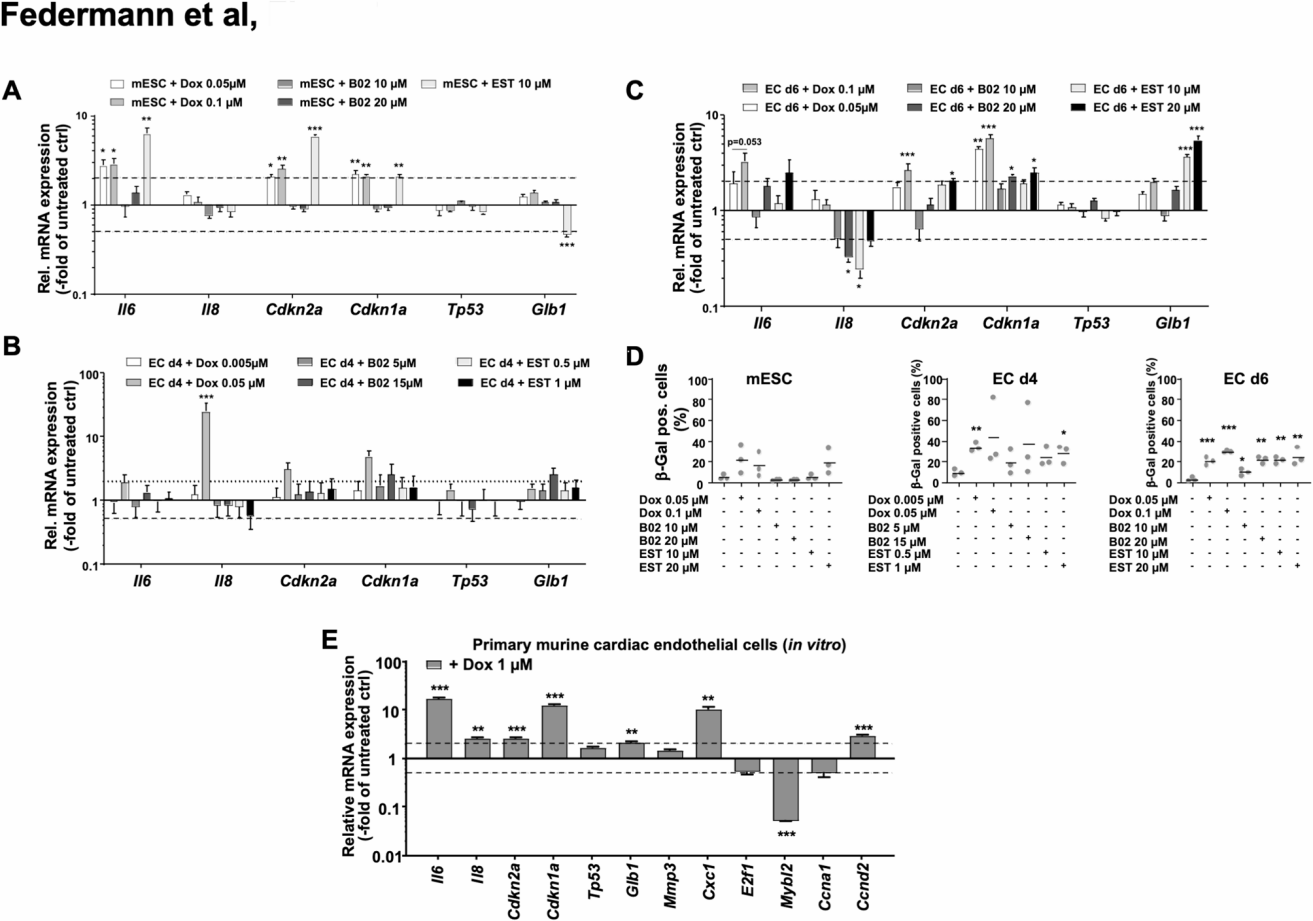
Differentiation dependent alteration in drug-induced mRNA expression of senescence-associated markers and β-Gal activity. **A-C**: Relative mRNA expression of a selected subset of senescence-related genes was analyzed in mESC, EC d6 (24 h + 24 h post incubation) and EC d4 (48 h) by RT-qPCR. Relative mRNA expression of genes in the corresponding untreated control was set to 1.0. Data represent the mean ± SEM of triplicate determinations. Only changes in mRNA levels of ≥ 2.0 and ≤ 0.5 were considered as biological relevant (dashed lines). One-way ANOVA, Student’s t-test (mESC + EST): *, *p* ≤ 0.05; **, *p* ≤ 0.01 as compared to the respective untreated control. **D**: The percentage of beta-Gal positive cells was analyzed as a surrogate marker of senescence in mESC, EC d6 (24 h + 24 h post incubation) and EC d4 (48 h) after exposure to the indicated concentrations of Dox, B02 or EST as described in methods. Data shown were obtained from *n* = 3 independent experiments. Student’s t-test: *, *p* ≤ 0.05; **, *p* ≤ 0.01; ***, *p* ≤ 0.001 as compared to the respective untreated control. **E**: Relative mRNA expression of a selected subset of senescence-related genes was analyzed in primary murine cardiac endothelial cells treated with Dox (1µM) as described before [22]. Relative mRNA expression of genes in the corresponding untreated control was set to 1.0. Data represent the mean ± SEM of triplicate determinations. Only changes in mRNA levels of ≥ 2.0 and ≤ 0.5 were considered as biological relevant (dashed lines). Student’s t-test: *, *p* ≤ 0.05; **, *p* ≤ 0.01; ***, *p* ≤ 0.01 as compared to the respective untreated control

Multiple molecular mechanisms contribute to Dox-induced cytotoxicity, including mitochondrial damage [58, 59]. Therefore, membrane potential was analyzed via live-cell imaging of MitoTracker™ and TMRM stained cells. The data obtained show that the mitochondria of progenitor EC d4 are affected by all three substances, with 0.05 µM Dox causing the strongest drop in mitochondrial membrane potential (Fig. 6A). Mitochondrial membrane potential of terminally differentiated EC (EC d6) was also significantly reduced following Dox and B02 treatment, yet remained largely unaffected by EST (Fig. 6B). Gene expression analysis of a subset of detoxification and mitochondrial function-related genes revealed a minor increase in the mRNA expression of the antioxidative factors *Hmox1* and *Sod1* in Dox-treated EC d6 as compared to mESC (Fig. 6C). A prominent upregulation of the mRNA expression of the mitochondrial biogenesis regulatory factor *Pgc1a* was observed following Dox exposure of terminally differentiated EC d6 (Fig. 6C). Comparative analysis of Dox-simulated *Pgc1a*-mRNA expression in cells of varying stages of differentiation using higher Dox concentration revealed its upregulation in EC d4 and EC d6 (Fig. 6D). Treatment with EST increased *Pgc1a* mRNA levels in mESC and, tendentially, in EC d6, but not EC d4 (Fig. 6D), again pointing to a compromised response of EPC to HDAC inhibition. B02 treatment did not affect *Pgc1a* mRNA expression in any of the cell types under investigation (Fig. 6D). To further elucidate the influence of drug treatment on metabolism, the activation status of the protein kinases p70S6 kinase, AMP kinase alpha and GSK-3β kinase, which play key roles in the regulation of metabolic processes, was investigated. We found clear differentiation- and drug-dependent differences in the activation status of these kinases. For example, the basal protein level of phosphorylated p70S6K, which regulates pathways related to PI3-kinase and protein synthesis, was largely unchanged in all cell types (Fig. 6E). The basal level of p-AMPKα was reduced in EC d4 and EC d6 as compared to mESC, pointing to differentiation specific differences in pathways regulating energy homeostasis, while the basal level of p-GSK-3β, which inhibits glycogen synthesis, was slightly enhanced in EC d4 (Fig. 6E). Following drug treatment, the phosphorylation of p70S6K was largely reduced in EC d4 after Dox exposure, while both B02 and EST treatment increased the p-p70S6K level in EC d4. Of note, the opposite effect was observed in B02- or EST-treated differentiated EC d6 (Fig. 6E), showing substantial differences in the metabolic response of progenitor versus differentiated cells following treatment with DNA repair / DDR inhibitors. Similarly, multiple agent- and differentiation dependent variations in the expression of p-AMPKα and p-GSK-3β were observed, for instance increased p-AMPKα levels in EST-treated EC d4 and EC d6 cells (Fig. 6E). Since we found signs of oxidative stress and mitochondrial damage following drug treatment and having in mind that Dox is known as inducer of ferroptosis [60], we additionally analyzed the mRNA expression of the ferroptosis-related marker genes *Gpx4* and *Slc7a11*. We found a significant increase in the mRNA levels of *Slc7a11* after Dox treatment in EC d4 but not in mESC or EC d6 (Fig. 6F). Since SLC7A11 regulates the uptake of cysteine, thereby promoting glutathione biosynthesis, this finding points to an antioxidative and thus ferroptosis-suppressing effect specifically in EC d4. Having in mind that doxorubicin also triggers senescence [27], the mRNA expression of senescence-associated factors was analyzed as well. Both mESC, EC d4 and EC d6 cells revealed an upregulated mRNA expression of individual senescence-related factors, including *IL6*,* IL8*,* Cdkn1a* and/or *Cdkn2a*, following Dox treatment (Fig. 7A-C). EST and B02 also stimulated isolated senescence-related responses in an agent- and differentiation-state-dependent manner (Fig. 7A-C). In line with the mRNA expression data, an increase in the percentage of beta-Gal-positive cells was also observed in EC d4 and EC d6 cells exposed to Dox and EST (Fig. 7D). To confirm the relevance of data obtained from mESC-based model, further analyses were performed using mRNA samples originating from untreated and Dox (24 h) treated primary mouse endothelial cells [22]. The results obtained are comparable to those found in EC d6 (Fig. 7C, Supplementary Fig. 5B), for example, a significant upregulation (about 10-fold) of the SASP markers *Il6* and *Cxc1* as well as of *Cdkn1a* (Fig. 7E). Taken together, drug treatment influences ferroptosis- and senescence-associated mechanisms in EPC.

### Impact of drug treatment of differentiating EC d4 on the functionality of differentiated EC d6

**Fig. 8:**
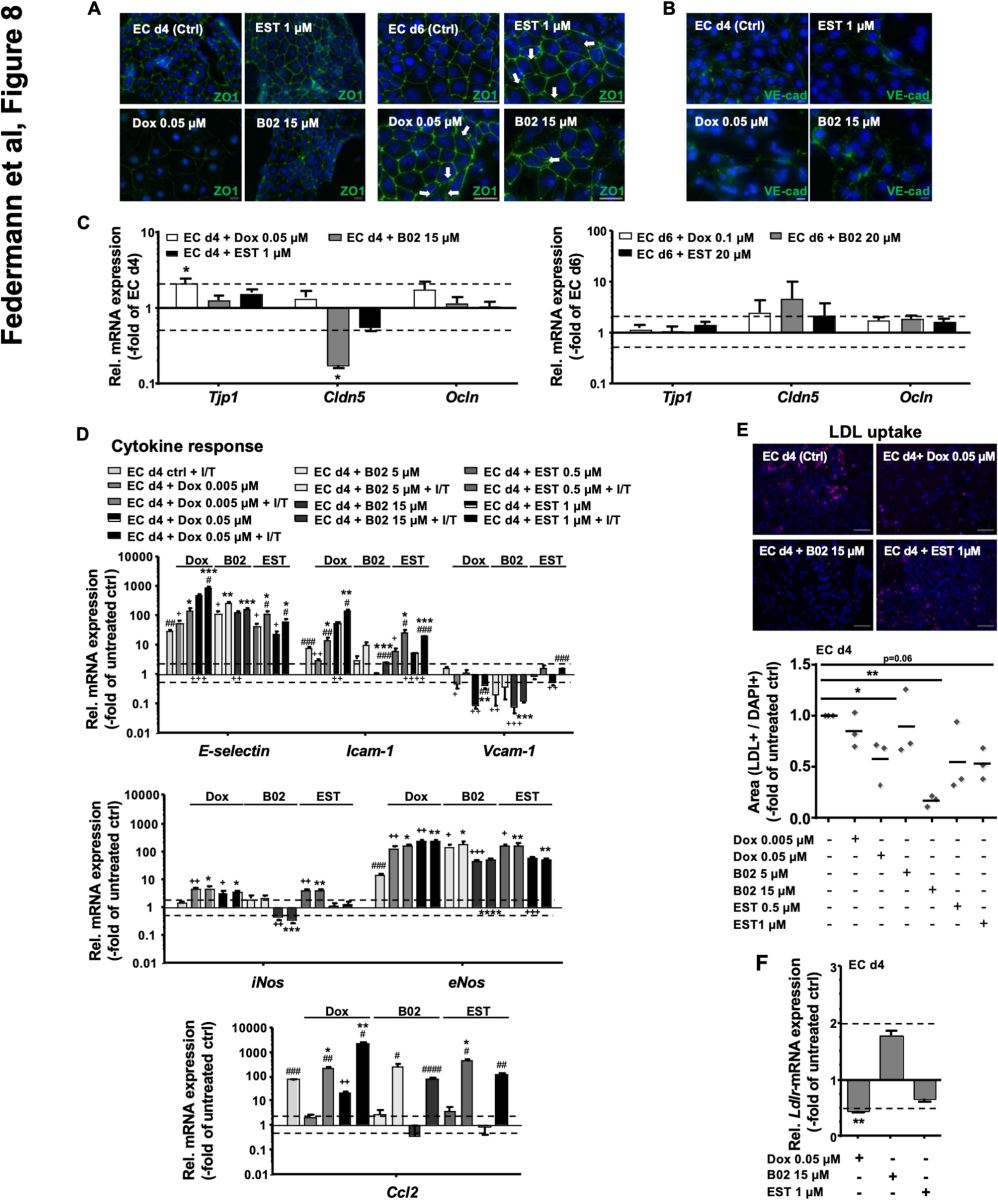
Treatment of EC d4 continuing differentiation leads to functional impairments of differentiated EC d6 progeny. **A**, **B**: Representative immunocytochemical staining of intracellular tight junction protein ZO1 (A) and (B) adherens junction protein VE-cadherin (VE-cad) following treatment of EC d4 and EC d6 with Dox and pharmacological inhibitors (see Fig. 1A). White arrows indicate gaps within the cell cluster. Scale bar: 10 μm. **C**: mRNA expression analysis of tight junction complex genes (*Tjp1*,* Cldn5*,* Ocln*) in EC d4 and EC d6 after drug exposure. EC d4 and EC d6 were treated with Dox, B02 and EST as described before (Fig. 4A treatment scheme). Relative gene expression of the corresponding untreated control was set to 1.0. Shown is the drug-induced fold-increase in mRNA expression as compared to corresponding untreated control group. Only changes in mRNA-expression ≥ 2.0 and ≤ 0.5 were considered as biologically relevant (dashed lines). Student´s t-test: *, *p* ≤ 0.05 as compared to untreated control. **D**: EC d4 were treated during ongoing differentiation for 48 h with 2 concentrations of Dox, EST or B02 before analyses were performed in EC d6. EC d6 were either left untreated or were exposed to pro-inflammatory cytokines (IL-1β / TNFα (+ I/T) (10 ng / ml, 1 h)) followed by mRNA expression analysis of the prototypical EC-related genes *E-Selectin*,* Vcam-1*,* Icam-1*,* Ccl2*,* eNos and iNos*. Relative mRNA expression in untreated EC d4 control was set to 1.0. Data represent the mean ± SEM of three independent experiments each performed in biological duplicates. The dashed lines indicate changes in mRNA levels of ≥ 2.0 and ≤ 0.5, which are considered as biologically relevant. Student‘s t–test: *, *p* ≤ 0.05; **, *p* ≤ 0.01; ***, *p* ≤ 0.001 as compared to EC d4 ctrl + I/T (*), treated vs. untreated (^+^), + I/T vs. – I/T (^#^). **E**: Immunocytochemistry-based analysis of acetylated LDL (magenta) uptake in EC d6 differentiated from drug-treated EC d4. For quantification, the LDL positive area was calculated in relation to the DAPI counterstained nucleus (blue). Single data points are shown as well as the mean of three independent experiments. Data are normalized to the untreated control which was set to 1.0. One-way ANOVA: *, *p* ≤ 0.05; **, *p* ≤ 0.01 as compared to untreated control. Scale bar: 50 μm. **F**: RT-qPCR gene expression analysis of *Ldlr mRNA expression* following Dox, B02 and EST treatment of EC d4 for 48 h. Relative *Ldlr*-mRNA expression in untreated control was set to 1.0. Data show the mean ± SEM from two independent experiments each performed in biological duplicates. Dashed lines indicate changes in mRNA expression of ≥ 2.0 and ≤ 0.5 and are considered as being biologically relevant. Student‘s t–test: **, *p* ≤ 0.01 as compared to untreated control

Apart from affecting proliferation, triggering cell death or influencing the differentiation process, drug treatment of EPC with subtoxic drug concentrations may also lead to a dysfunction of the surviving differentiated endothelial progeny. To scrutinize this hypothesis, EC d4 were treated with Dox or DDR/DNA repair inhibitors followed by the analysis of surrogate markers of prototypical endothelial functions in the differentiated progeny. Against the background of the physiologically highly important barrier function of the endothelium, the expression and localization of the tight junction protein ZO1 was analyzed by immunocytochemistry. Endothelial cells (EC) originating from untreated EC d4 showed homogenous structures of ZO1 containing tight junctions (Fig. 8A). By contrast, EC d6 that were differentiated from Dox-treated EC d4 revealed fragmented / interrupted ZO1 containing tight junctions (Fig. 8A), indicating that Dox impairs the differentiation fidelity of EPC. Treatment of EC d4 with DDR inhibitors B02 and EST did not affect the presence and assembly of the ZO1 protein into tight junctions (Fig. 8A). For additional control, terminally differentiated ECs (EC d6) were treated with the three substances and ZO1 assembly was analyzed 2 days later. The results of this experiment showed clearly visible gaps between ZO1-containing areas both after Dox, B02 and EST treatment (Fig. 8A), indicating disturbed ZO1-related tight barrier functions resulting from all drug treatments in differentiated EC d6. The morphological effects observed in EC d4 cells after Dox treatment are paralleled by a moderately increased ZO1 (*Tjp1*) mRNA expression as related to the untreated EC d4 control (Fig. 8C). Analyzing the mRNA expression of the ZO1 interacting factors Claudin 5 (*Cldn5*) and Ocludin (*OcIn*), B02 treatment reduced *Cldn5* mRNA expression in EC d4, but not in EC d6, as compared to the corresponding untreated controls. By contrast, Dox or EST exposure did not affect the mRNA expression of *Cldn5* and *OcIn* (Fig. 8C). For additional control, the formation of cell-cell junctions involving VE-cadherin was analyzed. As monitored by immunohistochemistry-based method, VE-cadherin protein expression and localization within the cytoplasm were disturbed in EC d6 differentiated from Dox-treated EC d4 (Fig. 8B). VE-cadherin containing cell-cell junctions were fragmentated with VE-cadherin being distributed throughout the cytoplasm. B02 and EST treatment of EC d4 did not trigger defects in VE-cadherin-related cell-cell junction formation in EC d6. Summarizing, the data show that drug treatment of EPC can influence the integrity of the endothelial barrier, which is partially due to changes in the expression of endothelial cell-cell adhesion factors.

Next, the response of EC d6 that have been differentiated from untreated or drug-treated EC d4 cells to pro-inflammatory cytokines was investigated. EC d6 derived from non-treated EC d4 responded to treatment with the pro-inflammatory cytokines IL-1β and TNFα with an upregulation of the mRNA expression of adhesion molecules (*E-selectin*,* Icam-1*), endothelial NO-synthase (*eNos*) and the chemokine CCl2 (*Ccl2*) (Fig. 8D), reflecting a healthy endothelial-like phenotype. Dox treatment of EC d4 resulted in a profound increase in the basal mRNA expression of the adhesion factors *E-selectin* and *Icam1* and a decreased *Vcam1* gene expression in EC d6 (Fig. 8D). Stimulation of EC d6 derived from Dox-treated EC d4 cells with IL-1β and TNFα potentiated the mRNA expression of the aforementioned endothelial genes (Fig. 8D). Additionally, treatment with pharmacological inhibitors caused similar changes in the basal mRNA expression of adhesion molecules (Fig. 8D). By contrast, basal mRNA expression of the chemokine *Ccl2* was increased following treatment with Dox, but remained unaffected by B02 or EST. Notably yet, IL-1β and TNFα treatment resulted in a further substantial upregulation of *Ccl2* gene expression in EC d6 that were differentiated from drug-treated EC d4 (Fig. 8D). Moreover, after Dox and EST exposure of EC d4, the resulting EC d6 showed elevated basal mRNA expression of *Nos* (*iNos*,* eNos*) as compared to the control (Fig. 8D). By contrast, treatment with B02 caused downregulation of basal *iNos* mRNA expression, while *eNos*-mRNA level was upregulated. Additional cytokine stimulation of differentiated EC d6 obtained from drug exposed EC d4 did not affect the mRNA expression levels of *Nos* in any of the cases (Fig. 8D). Moreover, receptor-mediated uptake of low-density lipoprotein (LDL) was comparatively investigated in EC d6 generated from untreated and drug-treated EC d4. Following Dox and inhibitor treatment of EC d4, a noticably reduced LDL uptake was observed in the differentiated EC d6 progeny, with B02-exposure of EC d4 revealing the strongest functional defect in the differentiated progeny (Fig. 8E). This functional impairment is largely independent of drug-induced changes in LDL receptor (*Ldlr)* mRNA expression of EC d4 cells as compared to the untreated ECd4 control (Fig. 8F). Taken together, the data demonstrate that drug exposure of EPC causes multiple functional alterations in differentiated EC d6.

## Discussion

To comparatively analyze drug-induced stress responses of mESC and thereof derived differentiating and terminally differentiated progeny, cell viability was comparatively analyzed up to 72 h after Dox treatment. Additionally, the inhibitor B02, which inhibits the HR-related repair protein RAD51 [42] and influences replicative stress responses [43, 61] and the HDAC class I inhibitor entinostat (EST) that interferes with multiple mechanisms of DSB repair and DDR [44, 45], were employed. The data obtained demonstrate cross-sensitivity of mESC to Dox and EST, indicating that both compounds affect cell viability of mESC by overlapping molecular mechanisms that are related to DNA damage formation and/or repair. By contrast, no cross-sensitivity was found for B02. Apparently, sole inhibition of RAD51-related functions is inefficient to trigger substantial cytotoxicity in mESC. Noteworthy, EPC (i.e., EC d4) revealed a significantly higher Dox and EST susceptibility as compared to both mESC and EC d6. This finding points to the existence of a so far unknown drug-sensitive time window during differentiation. The molecular mechanisms involved may be related to complex differentiation-related changes in chromatin structure that affect the accessibility of the DNA to chemicals, disturbed mitochondrial homeostasis or alterations in the expression or activity of drug targets. Of note, increased sensitivity of EC d4 cells was not observed for B02. Dox sensitivity of mESC and EC d6 was similar under situation of permanent treatment, which is in line with data obtained upon short-time Dox pulse-treatment [35]. However, Dox sensitivity varies between pulse-treatment and permanent treatment, showing that treatment regimen (e.g. high-dose short-time versus low-dose long-term treatment) are highly important to consider for meaningful hazard assessments in toxicological drug testing aiming to predict developmental toxicity.

Next, we asked the question whether drug exposure of differentiating EC d4 impacts their differentiation into EC d6. We found that Dox treatment of EC d4 influences the expression of both stem cell- and differentiation-related marker genes in EC d6 cells. Apparently, continuous exposure of EPC to Dox significantly impacts the mRNA expression of pluripotency- and EC differentiation-related maker genes in the differentiated EC d6 progeny, pointing to perturbations in EPC differentiation. Again, this novel finding is of high relevance in the context of drug hazard assessment in developmental toxicology. Since differentiation is accompanied by epigenetic alterations, we exemplarily monitored the acetylation status of nuclear histone proteins (H3, H4) and the cytosolic protein α-tubulin. We found that Dox selectively impacts the acetylation level of cytosolic α-tubulin but not of histone H3/H4 proteins. Moreover, the responsiveness of mESC, EC d4 and EC d6 to HDAC class I inhibitor EST and the RAD51 B02 inhibitor fluctuates with differentiation in an agent specific manner. We conclude that Dox and inhibitors of DNA repair / DDR influence epigenetic processes going along with development, which might be due to differentiation-dependent alterations in the expression of drug targets (e.g., HDAC, HAC). The selective impact of Dox on the acetylation level of cytosolic α-tubulin but not of H3/H4 indicates that Dox specifically influences the activity of the HDAC6 isoform, which has been reported to be upregulated by Dox [62]. Furthermore, HDAC6 inhibition reverses long-term cognitive dysfunction induced by Dox [63] and protects cardiomyocytes against Dox-induced acute damage by improving α-tubulin acetylation which, in turn, stabilize mitochondrial membrane potential [62]. Thus, HDAC6 is suggested as an epigenetically relevant molecular target of Dox-mediated effects on endothelial differentiation.

Given the high Dox sensitivity of EC d4, it is tempting to speculate that alterations in drug transport may be involved. Measuring Dox transport activity, we found that Dox import is particularly high in EC d4. Yet, at the same time, Dox efflux was also highest in EC d4. Thus, following an efficient drug uptake of Dox, mechanisms appear to be triggered in EC d4 that in turn promote an effective removal of this drug from these cells, possibly in a MDR1 dependent manner. Discussing the relevance of specific importers and exporters is difficult having in mind that the activity of multiple transporters defines the intracellular steady concentration of Dox. Since mRNA expression data of drug transporters did not match with protein expression data, we assume that fluorescence-based analysis of Dox transport activity provides the most meaningful results. Of note, having in mind that EC d4 cells reveal a higher Dox sensitivity than mESC and EC d6, drug transport does not provide a convincing molecular explanation for their Dox hypersensitivity. Monitoring the formation and removal of drug-induced DNA damage by measuring the steady-state level of nuclear γH2AX/53BP1 foci and the level of DNA single-strand breaks by use of the alkaline comet assay, we hypothesize that DSB repair by HR declines with the differentiation process, while NHEJ increases [64], which likely rests on transcriptional reprogramming. Analyzing time kinetics of DSB repair following exposure to a single dose of ionizing radiation (IR), we found that the overall DSB repair capacity does not majorly change during differentiation. Rather the preferred pathway of DSB repair shifts from an error-free DSB repair by HR to a more error-prone DSB repair by NHEJ, which is accompanied by changes in the morphology of repair-associated nuclear foci. We hypothesize that the observed changes in DSB repair activity and the morphology of nuclear repair foci results from differentiation-related changes in chromatin structure, lineage commitment and/or gene expression, which is in line with previous report [65]. Moreover, we found that EC d4 are characterized by a lower drug-induced formation of SSB as compared to mESC. Here, base excision repair (BER) is likely involved since the expression of BER factors in known to change with differentiation [66–68].

Having in mind that DNA damage interferes with replication fork progression, S-phase activity was investigated. Here, Dox evoked the most prominent abrogation of EdU incorporation in mESC. Analyzing replication fork progression by employing the DNA fiber spreading assay remarkable drug- and differentiation-dependent effects were found. We hypothesize that the differences observed are related to replication-specific characteristics of mESC, which are known to spend more time in S-phase than in gap phases and, among others, have a compromised G1/S checkpoint [69]. Moreover, factors involved in the regulation of DNA replication and replicative stress response are highly abundant in mESC. Thus, mESC experience enhanced replication stress under basal condition, but at the same time they extensively use protective mechanisms such as fork slowing and fork remodeling to ensure genome maintenance during replication. With ongoing differentiation, replicative stress decreases and cells are spending more time in gap phases that enable improved template quality control before entering S-phase [69]. Data obtained from our analysis of DDR-related replicative stress responses in mESC support the hypothesis that replication-associated mechanisms regulated by the ATR/CHK1/p53 axis are preferentially stimulated in mESC following drug exposure.

To further characterize molecular mechanisms contributing to the effects observed, differentiation dependent alterations in the mRNA expression of pre-selected susceptibility related factors were analyzed. While *Top2a* expression remained unaltered during differentiation, *Top2b* mRNA levels were upregulated in EC d6 as compared to mESC and EC d4. Since Topoisomerase IIβ is involved in transcriptional regulation [70] and, moreover, is present in mitochondria [71], its upregulation is indicative of alterations in the transcriptome and/or the metabolic shift from glycolysis towards oxidative phosphorylation (OxPhos) that goes along with differentiation [72, 73]. Accordingly, we assume that Dox-mediated inhibition of Top2β accounts for some of the effects observed, especially with respect to mitochondria. Moreover, transient changes in the mRNA expression of antioxidative defense-related factors Nos3 and Nrf2 were observed. Transient fluctuations of antioxidative functions were also reported during renal differentiation of hiPSC [74]. Discussing the potential impact of Dox-induced formation of ROS caused by mitochondrial damage, it is important to note that ROS originating from mitochondria do not reach the nucleus and, therefore, are likely not relevant for the activation of the DDR [75]. In addition, we found down-regulated mRNA expression of HR-related DSB factors (i.e., Brca1, Rad51) with ongoing differentiation and, moreover, also upon drug exposure in cells of all differentiation stages. These findings, together with the data obtained from the analysis of DSB repair of IR-induced DSB are in line with literature, demonstrating a shift from error-free DSB repair by HR in stem cells to more error-prone DSB repair by NHEJ in the differentiated progeny [76–78]. On the protein level, we found RAD51 and CHK1 to be downregulated after high dose EST treatment in EC d6 only. This observation may reflect the higher abundance of replication stress- and HR-related factors in fast replicating stem and progenitor cells. Notably, class I HDACi also downregulate the mRNA and protein expression of DSB repair and DDR-related factors in malignant cells [79], which provides a molecular basis for their anticancer efficacy, especially in combination with conventional anticancer drugs. Analysis of the activation status of DDR-related factors show that especially Dox-mediated activation of p53-related functions, which are known to change with development [80], are compromised in differentiating EC d4. In addition, Dox-induced activation of the heterochromatin regulatory factor Kap1 (TIF1β) was specifically observed in mESC. Interestingly in this context, p-KAP1 (S824) contributes to the maintenance of stemness in ESC [81]. Of note, treatment with EST, but not B02, also increased p-KAP1 (S824) protein level in mESC, supporting the hypothesis of an interplay between epigenetic mechanisms and DDR and DNA repair [44, 56]. Overall, the data demonstrate highly selective and differentiation-dependent effects of DDR/DNA repair inhibitors on distinct mechanisms of the DDR. Overall, the data strongly indicate that transcriptional changes in the expression of TopoII isoforms, antioxidative functions, DSB repair-related factors as well as alterations in ATM/ATR-related post-translational mechanisms of the DDR contribute to the observed changes in drug responsiveness in relation to the differentiation stage.

Analyzing mitochondria-related functions we found substantial mitochondrial damage caused by Dox treatment, which is in line with literature [58, 59]. Mono-treatment with DNA repair / DDR inhibitors also evoked mitochondrial damage, though to a lower extent than Dox, which is likely due to impaired repair of ROS-related mitochondrial DNA damage occurring under basal situation. The results demonstrate a so far unknown pronounced susceptibility of mitochondria of differentiating cells to substances interfering with genomic stability. We hypothesize that this is related to changes in mitochondrial homeostasis and metabolic activity going along with differentiation [73] and/or mitotoxic effects of metabolites that are endogenously generated in varying quantities and/or qualities depending on the cellular differentiation status. This hypothesis is in line with the observed profound differentiation- and drug-dependent variations in the activation status of the metabolism-associated protein kinases p70S6K, AMPKα and GSK3β. Furthermore, we found a particular high mRNA expression of *Pgc1a*, which plays a key role in regulating mitochondrial biogenesis and is involved in maintaining mitochondrial homeostasis [82], in Dox-treated EC d6. Again, this finding may be related to an increase in OXPHOS with differentiation [72, 73] and, moreover, likely reflects cellular recovery from Dox-induced mitotoxicity. Taken together, the data demonstrate that mESC, EC d4 and EC d6 differently respond to Dox-, B02- or EST-induced damage with either a stimulation or inhibition of multiple mechanisms related to mitochondrial biogenesis, mitochondrial homeostasis and metabolism. Supporting the hypothesis of an interplay between DDR-related factors and mitochondrial functions [83] during endothelial differentiation. In addition, the significant increase in the mRNA levels of the ferroptosis-related cysteine transporter *Slc7a11* observed after Dox treatment of EC d4, points to a Dox-inducible antioxidative and thus ferroptosis-suppressing effect in EPC.

Stem cell-related erroneous or insufficient regeneration of injured tissue can explain developmental defects, accelerated ageing processes as well as chronic toxicity resulting from acute or long-lasting exposure to chemicals. Therefore, from a toxicological point of view, it is a highly important question whether exposure of EPC to subtoxic drug concentrations results in a sustained dysfunction of the surviving differentiated endothelial progeny. To address this aspect, cell-cell adhesion, LDL uptake and responsiveness to pro-inflammatory cytokines were monitored as surrogate markers of endothelial functionality. Dox exposure of EC d4 impairs the correct formation of ZO1 containing tight junctions, which are of relevance for endothelial barrier function [84], in the differentiated EC d6 progeny. Of note, ZO1-containing tight junctions can also be damaged in terminally differentiated EC d6 following treatment with Dox and DDR-modifying compounds. Thus, we hypothesize that Dox treatment of EC d4 disturbs the assembly of components of cell-cell adhesion complexes in terminally differentiated EC d6 by either transcriptional and/or post-transcriptional mechanisms. Moreover, treatment of EC d4 with all three substances promotes the development of a distinct pro-inflammatory phenotype in differentiated EC d6, which is further aggravated upon cytokine stimulation. In addition, Dox and pharmacological inhibitors of DNA repair/DDR influence posttranscriptional mechanisms regulating the formation, internalization and/or signaling of the LDL-receptor, eventually resulting in a defective uptake of LDL into EC d6. Alternatively, in consequence of a pro-inflammatory state of endothelial cells, LDL transport could also take place through a dysfunctional endothelial barrier as previously suggested for the process of atherosclerosis [85]. Thus, it´s rational to assume that the observed drug-induced alterations in inflammation- and SASP-associated cytokine production and signaling contributes to DDR-induced senescence and endothelial dysfunction. Taken together, we speculate that substances interfering with genomic stability disturb EPC lineage commitment and/or EPC differentiation accuracy, thereby eventually promoting the development of a dysfunctional differentiated EC progeny that is suffering from defects in endothelial barrier-related functionality, LDL uptake and inflammatory responses. We assume that drug-induced dysfunctions in differentiated endothelial-like cells resulting from EPC exposure are limited to individual endothelial functions, are agent-specific and, moreover, likely involve senescence-associated mechanisms related to Dox-stimulated DDR.

Summarizing our data and having in mind that EPC are known as relevant targets of Dox-induced cardiotoxicity [27], we suggest them as particular promising target for novel preventive concepts aiming to mitigate EPC injury caused by Dox-based anticancer regimen, thereby promoting heart regeneration. Exploiting EPC as drug targets has recently also been suggested by others [86], supporting the idea of protecting non-cardiomyocytes to improve regenerative processes in the heart [87]. Noteworthy in this context, embryonic stem cell-derived cardiomyocytes have been considered as useful for the treatment of Dox-induced cardiomyopathy [88–90]. The concept of chemoprotection of stem cells gets support by recent report showing that glutathione treatment of EPC is able to protect the heart from Dox-mediated cardiotoxicity [91]. In extension to this data, we hypothesize protection of EPC from Dox-induced DNA damage and/or inhibition of pro-toxic mechanisms of the DDR as useful strategy to improve the tolerability of anthracycline-based anticancer therapy.

## Conclusion

**Fig. 9:**
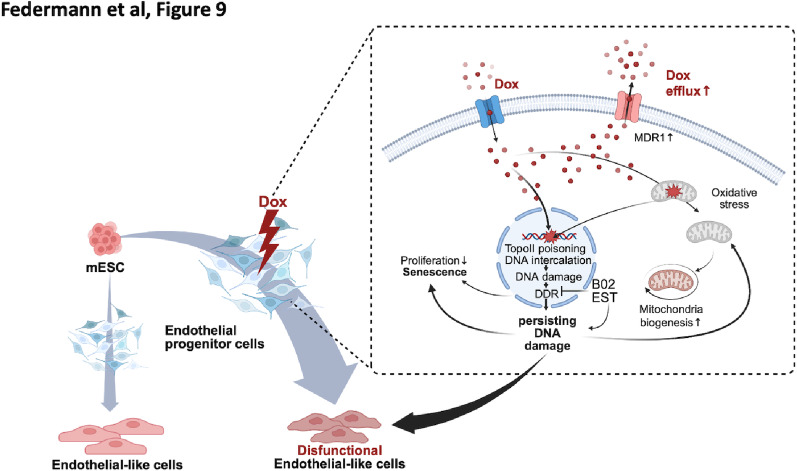
Graphical model: Dox-induced DNA damage in endothelial progenitor cells compromises the functionality of the differentiated EC progeny. Based on the data, we hypothesize that there is a Dox-vulnerable window during endothelial differentiation that impacts an error-free differentiation process of endothelial progenitor cells, thereby eventually promoting the development of a dysfunctional differentiated EC progeny. This likely involves differentiation dependent changes in gene expression, DNA repair and DDR capacity as well as mechanisms related to drug transport, metabolism and senescence. Pharmacological inhibition of DNA repair-related factors in endothelial progenitor cells by B02 and EST also promotes agent-specific functional impairments in the differentiated EC progeny, highlighting the key role of a robust basal DNA repair capacity in healthy endothelial development (created with BioRender.com)

The major aim of our study was to identify potential toxicological hazards resulting from anthracycline-based anticancer therapy with regard to developmental processes in pediatric patients and/or regenerative mechanisms of the adult heart. Since endothelial dysfunction is known to contribute to anthracycline-induced cardiotoxicity [24, 25], we hypothesize that Dox-induced DNA damage in drug-sensitive murine EPC (EC d4) disturbs a healthy further development of endothelial functions in terminally differentiated endothelial-like cells **(**Fig. 9**).** The identification of a transient drug-vulnerable time window during differentiation provides a feasible molecular basis to explain late cardiotoxicity resulting from anthracycline-based anticancer therapy. Of note, inhibition of mechanisms of DNA repair and DDR by the RAD51 inhibitor B02 or the HDACi EST also caused agent-specific impairments in the differentiation-dependent development of endothelial functions in the surviving progeny. These findings highlight the physiological relevance of an adequate basal and adaptive DNA repair and DDR capacity in differentiating EPC in order to counteract the development of a dysfunctional endothelium. Hence, we speculate that chronic cardiotoxicity observed in patients undergoing anthracycline-based treatment regimen may be partially driven by error-prone regenerative processes of a damaged surviving EPC population, eventually resulting in a compromised functionality of the differentiated endothelial progeny and imbalanced endothelial homeostasis. Correspondingly, we suggest pharmacological measures (e.g., dexrazoxane, inhibitors of pro-toxic mechanisms of the DDR, DSB repair activators etc.) aiming to specifically protect endothelial progenitor cells (EPC) from Dox-induced formation of DNA damage and related activation of deleterious DDR-related pathways as appropriate approach to preserve EPC´s fitness and differentiation accuracy, thereby promoting regenerative processes in the heart and, consequently, reducing the risk of late-onset cardiomyopathy resulting from Dox-based treatment regimen. The results obtained from our mESC-based in vitro models are of fundamental toxicological relevance regarding developmental and regenerative processes in adults and/or pediatric patients receiving Dox-containing chemotherapy and, moreover, are also of general relevance for toxicological testing, especially in developmental toxicology.

## Supplementary Information

Below is the link to the electronic supplementary material.

Supplementary Material 1.

Supplementary Material 2.

## Data Availability

No datasets were generated or analysed during the current study.

## Abbreviations

AMPKα: AMP-activated protein kinase alpha subunit
ATM: Ataxia telangiectasia mutated protein
Atp7a/b: ATPase, copper transporting, alpha/ beta polypeptide
ATR: Ataxia telangiectasia and Rad3-related protein
B02: RAD51 inhibitor B02
BER: Base excision repair
bFGF: Basic fibroblast growth factor
BM MSC: Bone marrow multipotent stromal cells
BMP4: Bone morphogenetic protein 4
Brca1: Breast cancer 1
CHF: Congestive heart failure
CHK: Checkpoint kinase
Ctr2: Copper transporter 2
DDR: DNA damage response
Dox: Doxorubicin
DSB: DNA double-strand breaks
EC/EC d6: Endothelial-like cells
EC d4: Endothelial progenitor cells
EST: Entinostat
Eto: Etoposide
Flk1: Fetal liver kinase-1
GPCR: G-protein coupled receptor
Gpx: Glutathione peroxidase
GSK-3β: Glycogen synthase kinase 3 beta
GSKi: Glycogen synthase kinase-3 inhibitor XVI
Gstm: Glutathione S-transferase, mu 1
HDAC: Histone deacetylase
hiPSC: Human induced pluripotent stem cells
Hmox1: Heme oxygenase (decycling) 1
HR: Homologous recombination
HU: Hydroxy urea
H2AX: H2A histone family, member X
IC: Inhibitory concentration
KAP1: KRAB-associated protein 1
LDL: Low density lipoprotein
Ldlr: Low density lipoprotein receptor
Mdr1: Multi drug resistance protein 1
mESC: Mouse embryonic stem cells
Mrp1: Multidrug resistance-associated protein 1
Nanog: Nanog homeobox
NHEJ: Non-homologous end-joining
NO: Nitric oxide
NOS: Nitric oxide synthases
Nqo1: NAD(P)H dehydrogenase [quinone] 1
Nrf2: Nuclear factor erythroid 2-related factor 2
Oct4: Octamer binding transcription factor 4
Oct2: Organic cation transporter 2
Pecam1: Platelet endothelial cell adhesion molecule-1
Pgc1a: Peroxisome proliferator-activated receptor gamma coactivator 1-alpha
ROS: Reactive oxygen species
RPA: Replication protein A
SC: Stem cells
Sod1: Superoxide dismutase
SSB: DNA single-strand breaks
Tie1: Tyrosine kinase with immunoglobulin-like and EGF-like domains 1
TOP2: Topoisomerase type II
VE-Cadherin: vascular endothelial cadherin
VEGF: Vascular endothelial growth factor
ZO1: Zonula occludens-1
53BP1: P53-binding protein 1
