# Supplementary material for: Endothelial progenitor cell susceptibility to DNA damaging and DDR-modulating compounds determines endothelial differentiation accuracy
Source: Stem Cell Res Ther. 2026 Jun 25;17:226. doi: 10.1186/s13287-026-05087-1 (PMC13308190; doi:10.1186/s13287-026-05087-1)
Supplement: Supplementary file 1 — Supplementary Material 1. [file 13287_2026_5087_MOESM1_ESM.pdf]

Supplementary figures – *Stem Cell Research and Therapy*

Progenitor cell´s susceptibility to DNA damaging and DDR-modulating compounds determines endothelial differentiation accuracy

Sina Federmann[1], Michelle Westerhoff[2], Andreas S. Reichert[2], Gerhard Fritz[1*]

[1]Institute of Toxicology, Medical Faculty and University Hospital, Heinrich-Heine-University Duesseldorf, Moorenstrasse 5, 40225 Duesseldorf, Germany;
[2]Institute of Biochemistry and Molecular Biology I, Medical Faculty and University Hospital, Heinrich-Heine-University Duesseldorf, Universitätsstrasse 1, 40225 Duesseldorf, Germany;

Correspondence
Gerhard Fritz, PhD
Institute of Toxicology
Medical Faculty and University Hospital
Heinrich-Heine-University Duesseldorf
Moorenstrasse 5, 40225 Duesseldorf, Germany
Phone: +49-211-8113022, Fax: +49-211-8113013
E-mail: fritz@uni-duesseldorf.de

Federmann et al, Supplementary Fig. 1

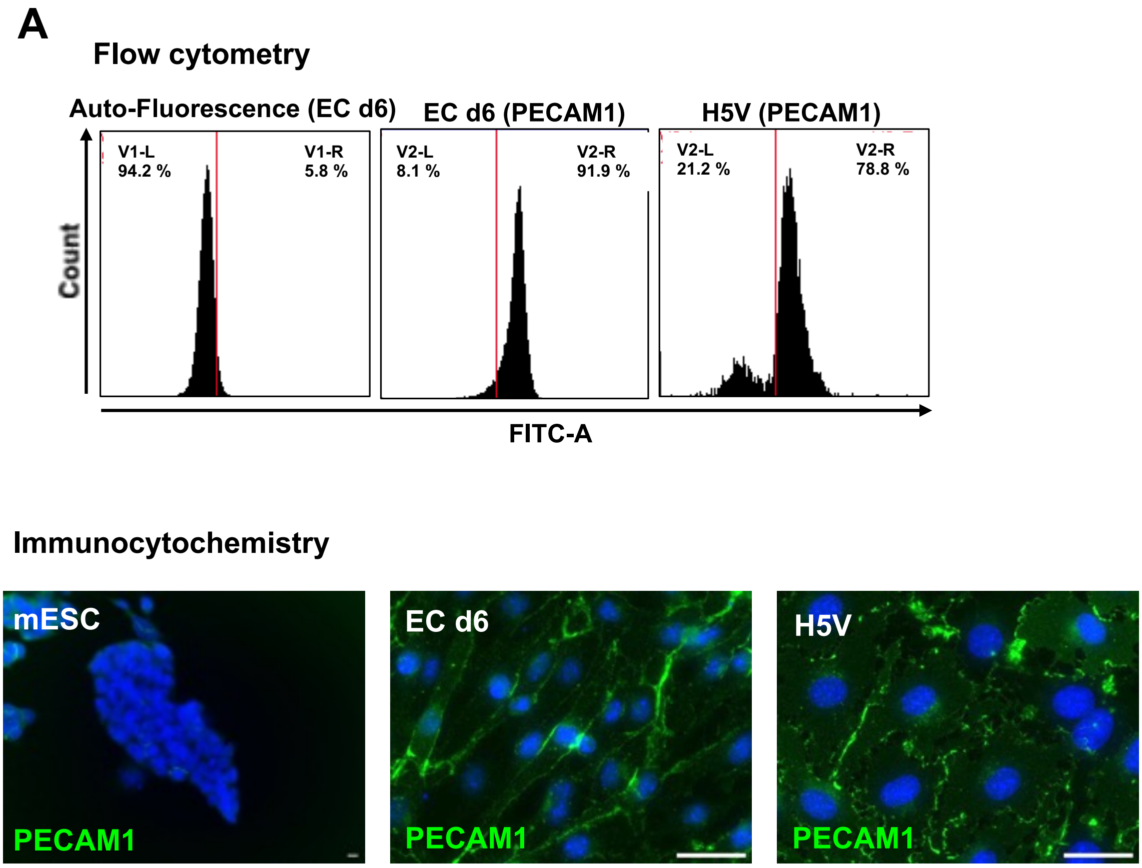

Supplementary Figure 1: Purity of mESC-derived EC d6 population.
A, B: The protein expression of endothelial marker protein PECAM1 was analyzed in EC d6 endothelial-like cells that were differentiated from mESC by flow cytometry-based analysis **(A)** and immuncytochemistry **(B)**. For control, PECAM1 protein expression was also investigated in murine H5V endothelial cell line.

Federmann et al, Supplementary Fig. 2

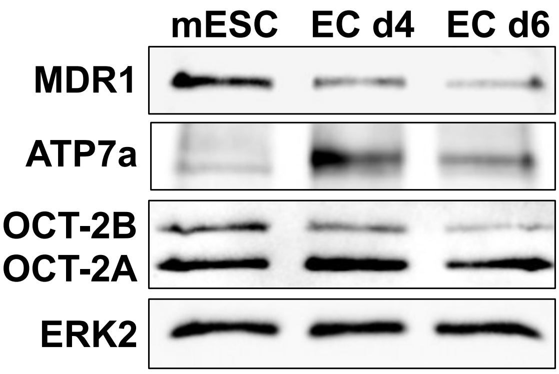

Supplementary Figure 2: Basal protein expression of drug transporters in mESC, EC d4 and EC d6.

Total protein extracts isolated from mESC, EC d4 and EC d6 cells were subjected to Western blot analysis to analyze the protein expression of drug transport-related proteins MDR1 (exporter), ATP7a and OCT-2 (importers) under basal situation.

Federmann et al., Supplementary Fig. 3

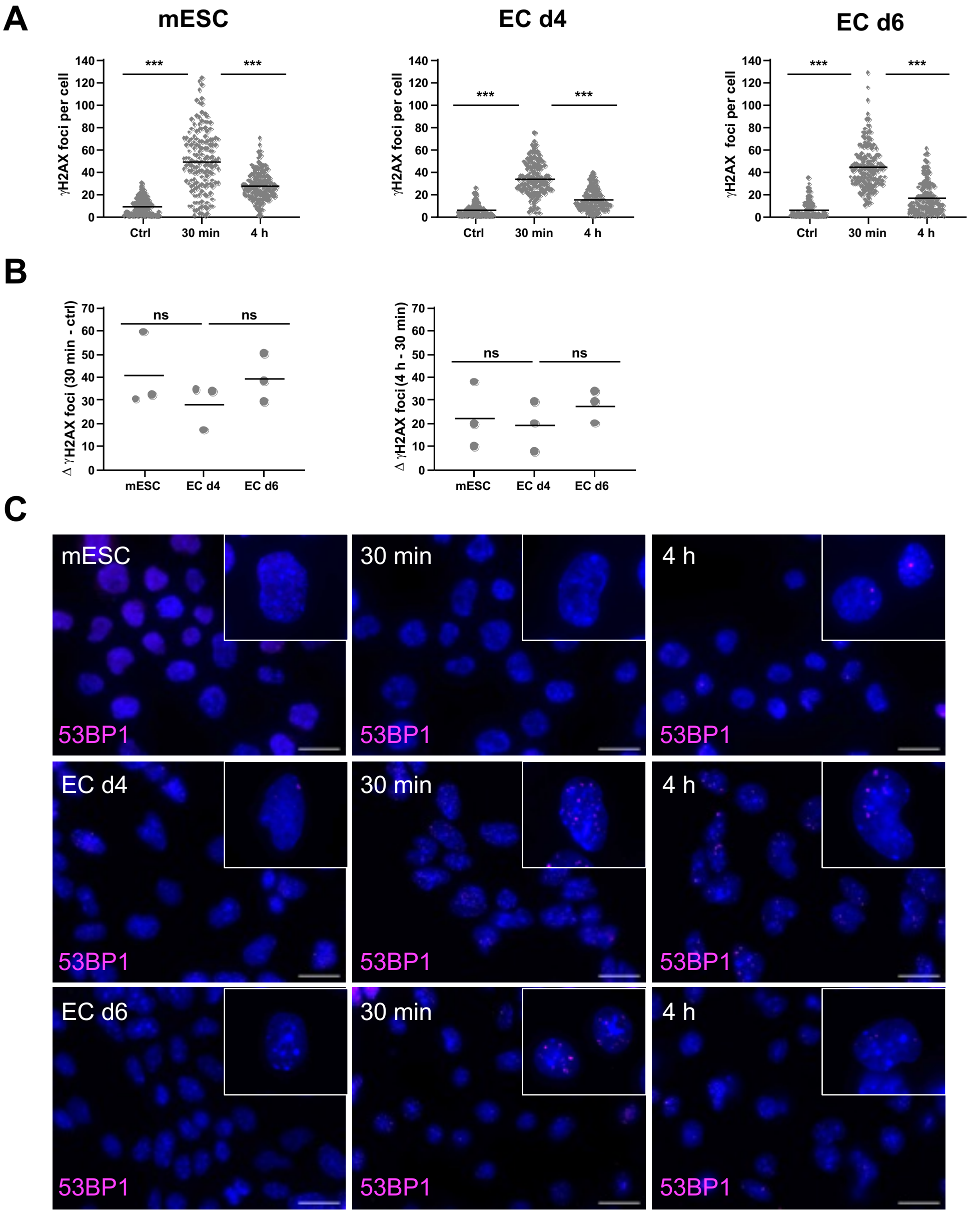

Supplementary Figure 3: DSB repair capacity of mESC, ECd4 and ECd6.

Cells in different states of differentiation (undifferentiated mESC, ECd4 and ECd6) were treated with a single dose of ionizing irradiation (IR, 2 Gy). After post-incubation period of 30 min and 4 h, the number of nuclear γH2AX and 53BP1 foci was analyzed.

A: Analysis of the number of nuclear γH2AX foci present in irradiated mESC, EC d4 and EC d6 at different time points after IR exposure as compared to corresponding non-irradiated controls. Data shown are from the analysis of 150 cells from three biological replicates (n=3, N=50 cells per experiment). One-way ANOVA: ***, $p \leq 0.001$.

B: Calculation of the time-dependent decrease in the number of nuclear γH2AX foci in cells of different differentiation state.

C: Analysis of the number of nuclear γH2AX foci present in irradiated mESC, EC d4 and EC d6 at different time points after IR exposure as compared to corresponding non-irradiated controls. Shown are representative pictures (scale bar: 20 µm).

Federmann et al, Supplementary Fig. 4

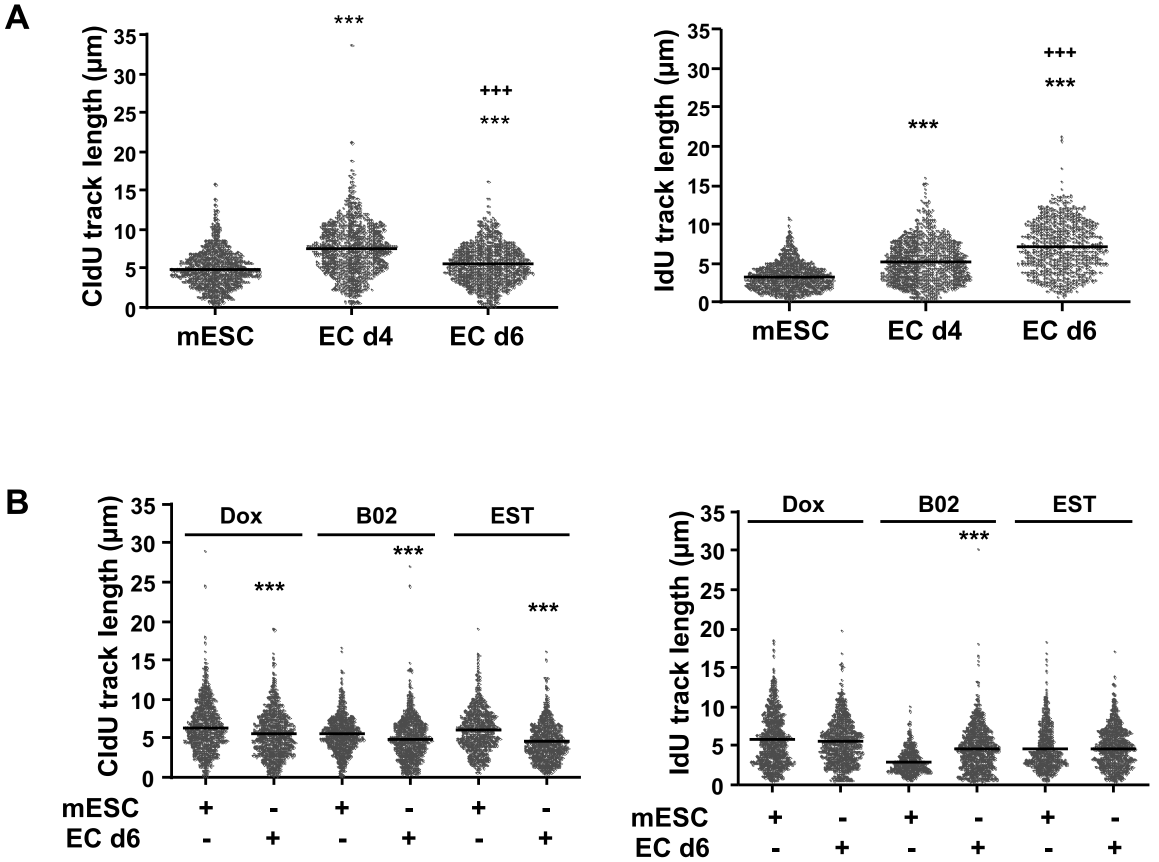

Supplementary Figure 4: The differentiation status impacts the replication fork progression under basal condition and after drug treatment.

A, B: Replication fork progression was analyzed by use of the DNA fiber spreading assay as described in methods. Data shown were obtained from n=3-4 independent experiments with 200 fibers being analyzed per experimental sample (see Figure 4). (A) Track length of CldU (left) and IdU (right) of untreated mESC, EC d4 and EC d6 was analyzed comparatively. One-way ANOVA: ***, $p \leq 0.001$ as compared to mESC; $^{+++}$, $p \leq 0.001$ as compared to EC d4. (B) Cells were treated undifferentiated and terminally differentiated on day 6 for 24 h with an equimolar dose of Dox, B02 and EST. The length of CldU-(left) and IdU-tracks (right) of treated mESC and EC d6 were analyzed comparatively. Student's t-test: ***, $p \leq 0.001$ as compared to mESC.

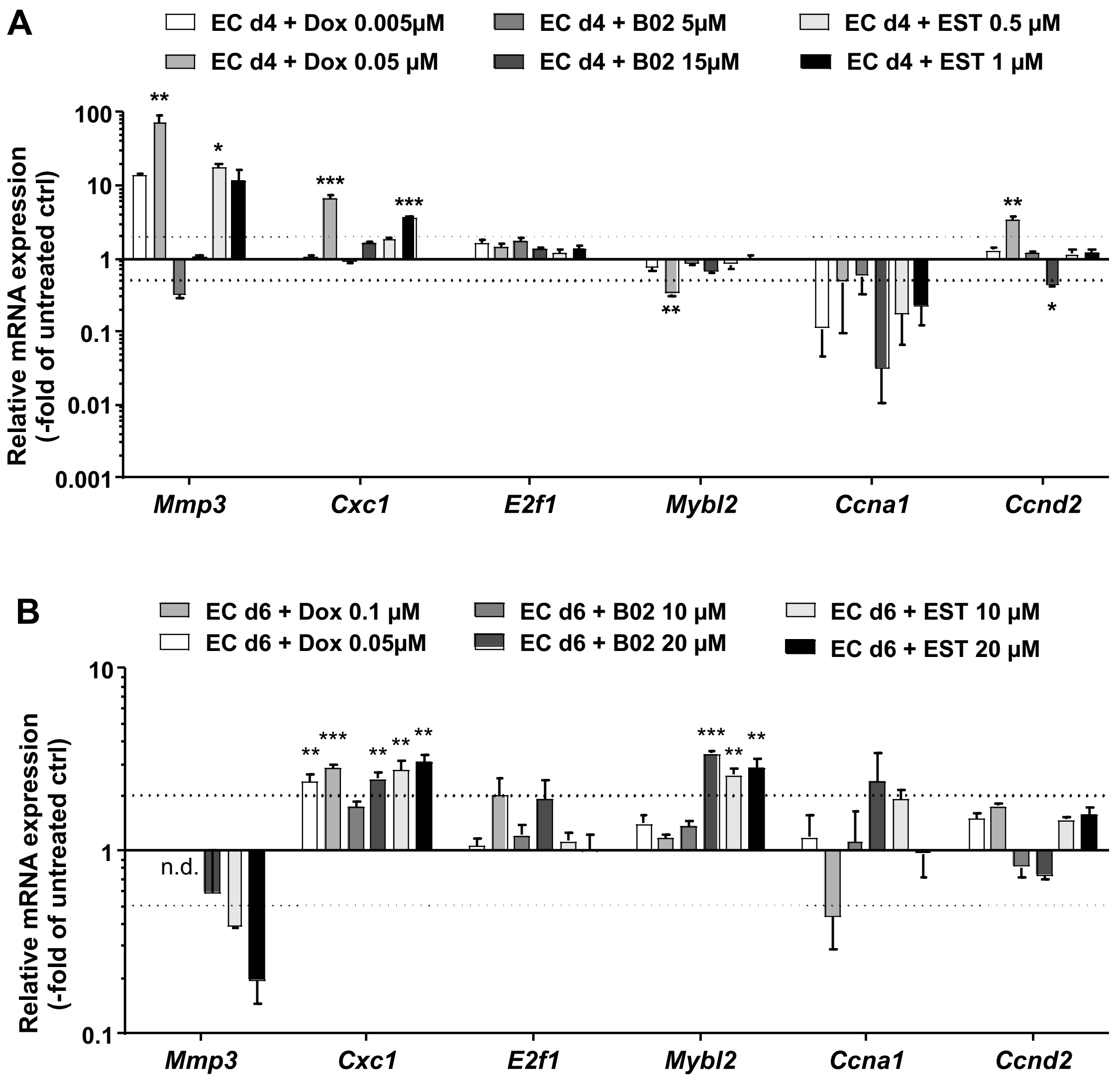

Supplementary Figure 5: Differentiation dependent alteration in drug-induced mRNA expression of senescence-associated markers.

A, B: Relative mRNA expression of a selected subset of senescence-related genes was analyzed in EC d4 (48 h) (A) and EC d6 (24 h + 24 h post-incubation) (B) after exposure to the indicated concentrations of Dox, B02 or EST by RT-qPCR. Relative mRNA expression of genes in the corresponding untreated control was set to 1.0. Data represent the mean ± SEM of triplicate determinations. Only changes in mRNA levels of ≥ 2.0 and ≤ 0.5 were considered as biological relevant (dashed lines). Student's t-test: *, $p \leq 0.05$; **, $p \leq 0.01$ as compared to the respective untreated control.
