# Supplementary material for: Endothelial progenitor cell susceptibility to DNA damaging and DDR-modulating compounds determines endothelial differentiation accuracy
Source: Stem Cell Res Ther. 2026 Jun 25;17:226. doi: 10.1186/s13287-026-05087-1 (PMC13308190; doi:10.1186/s13287-026-05087-1)
Supplement: Supplementary file 2 — Supplementary Material 2. [file 13287_2026_5087_MOESM2_ESM.pdf]

Progenitor cell´s susceptibility to DNA damaging and DDR-modulating compounds determines endothelial differentiation accuracy

Sina Federmann[1], Michelle Westerhoff[2], Andreas S. Reichert[2], Gerhard Fritz[1*]

[1]Institute of Toxicology, Medical Faculty and University Hospital, Heinrich-Heine-University Duesseldorf, Moorenstrasse 5, 40225 Duesseldorf, Germany;

[2]Institute of Biochemistry and Molecular Biology I, Medical Faculty and University Hospital, Heinrich-Heine-University Duesseldorf, Universitätsstrasse 1, 40225 Duesseldorf, Germany;

Correspondence

Gerhard Fritz, PhD

Institute of Toxicology

Medical Faculty and University Hospital

Heinrich-Heine-University Duesseldorf

Moorenstrasse 5, 40225 Duesseldorf, Germany

Phone: +49-211-8113022, Fax: +49-211-8113013

E-mail: fritz@uni-duesseldorf.de

Supplementary table 1: Primer sequences for RT-qPCR mRNA expression analyses.

Target		NCBI reference sequence	Sequence (5'-3')	
Abcc1 (Mrp1)	ATP Binding Cassette Subfamily C Member 1, Multidrug Resistance Associated Protein 1 (MRP1)	NM_001425178	fwd	GTGACTCTCAAGGGCTCCG
β-Actin	Actin, beta	NM_007393	fwd	GCATTGCTGACAGGATGCAG
			rev	CCTGCTTGCTGATCCACATC
Atp7a	ATPase copper transporting alpha	NM_001109757.2	fwd	ACCACCATCGCATTTGCCTA
			rev	GGAGGTTTTGCCCTTCGCTA
Atp7b	ATPase copper transporting beta	NM_007511.3	fwd	AAGAGGCCAGTCGGAAAATCT
			rev	CCCCCTTCGTAGCCAACATT
Brca1	BRCA1 DNA repair associated	NM_009764	fwd	TTGTGAGCGTTTGAATGA
			rev	ACCTGGCTTAGTTACTGT
Ccl2	Chemokine (C-C motif) ligand 2	NM_011333	fwd	ATGAGATCAGAACCTACAACT
			rev	TCCTACAGAAGTGCTTGAG
Ccna1	Cyclin A1	NM_007628.4	fwd	GGAAATTGCAGCTTGTCGGG
			rev	CGGTCGATGGGGGATACTGA
Ccnd2	Cyclin D2	NM_009829.3	fwd	AAGGAGGTAAGGGAAGCACTC
			rev	TGGGGCTTCACAGAGTTGTC
Cdkn1a (p21)	Cyclin dependent kinase inhibitor 1A (p21)	NM_001040654	fwd	AAAGCGAACTCGAGGAGAGC
			rev	TCATCATCACCTGAATCGGGG
Cdkn2a (p16)	Cyclin dependent kinase inhibitor 2A (p16)	NM_007669	fwd	ACCTGAATAGCACTTTGGAAA
			rev	TCTGAGCAATGTCAAGAGTC
Chk1	Serine/Threonine-Protein Kinase Chk1	NM_001410205	fwd	GTGCCTATGGAGAAGCCAGAC
			rev	CCGAAATACCGTTGCCAAGC
Cldn5	Transmembrane, tight junction protein claudin 5	NM_013805.4	fwd	AGTTAAGGCACGGGTAGCAC
			rev	GTACTTCTGTGACACCGGCA
Ctr2	Cationic Amino Acid Transporter 2	NM_001044740	fwd	GTGCAGTGGCAGCTGTGAT
			rev	AGCCAGGTTGGTACCTGAGA
Cxc1	C-X-C motif chemokine ligand 1	NM_008176.3	fwd	TGCACCCAAACCGAAGTCAT
			rev	ACTTGGGGACACCTTTTAGCAT
E2f1	Transcriptionfactor E2F1	NM_007891.5	fwd	AGCCTAGGGATTCAGGGTGT
			rev	CCCACCCTTTGCTTATCCCC
E-selectin	Selectin, endothelial cell	NM_011345	fwd	TGCGAGAAGAACGGATAGA
			rev	CTGAATTGCCACCAGATGT
Flk1	Fetal liver kinase 1	NM_001363216	fwd	GTTCTGCGTGGAGACCCGAGC
			rev	CAAAGCCAGTCCAGGTCCCGC
Gapdh	Glyceraldehyde-3-phosphate dehydrogenase	NM_008084	fwd	GACGGCCGCATCTTCTTGTGC
			rev	CACTGCAAATGGCAGCCCTGG
Galb1	Beta-galactosidase	NM_009752	fwd	CGGATACCCCGCTTCTACTG
			rev	AGGGCACGTACATCTGGATAG
Gpx1	Glutathione peroxidase 1	NM_008160	fwd	TTGGTGATTACTGGCTGC
			rev	TGATATTCAGCACTTTATTCTTAGTAG
Gpx4	Glutathione peroxidase 4	NM_008162.4	fwd	CTGGGAAATGCCATCAAAT
			rev	GTCCTTCTCTATCACCTGG
Gstm1	Glutathione S-Transferase Mu 1	NM_010358	fwd	ACACAGCCTTCATTCTCC
			rev	AATTCTAGGAAGCGTGAGTT
Hmox1	Heme oxygenase (decycling) 1	NM_010442	fwd	CCAGAGTCCCTCACAGAT
			rev	CCCAAGAGAAGAGAGCCA

Icam-1	Intercellular adhesion molecule 1	NM_010493	fwd	TGCTCAGGTATCCATCCAT
			rev	GGAAACGAATACACGGTGAT
Il6	Interleukin 6	NM_031168	fwd	AGTTGCCTTCTTGGGACTGA
			rev	CAGAATTGCCATTGCACAAC
Il8	Interleukin 8	NM_011339	fwd	CATCTTCGTCCGTCCCTGTG
			rev	CCTTCACCCATGGAGCATCA
Ldlr	Low density lipoprotein receptor	NM_001252658.1	fwd	CCAATCGACTCACGGGTTCA
			rev	ACAGTGTCGACTTCTCTAGGC
Mdr1 (Abcb1a)	Multi drug resistance protein 1	NM_011076.3	fwd	TGGAACTTGAAGAGGACC
			rev	GCATAACGAAACATTGTAAGC
Mmp3	Matrix metallopeptidase 3	NM_010809	fwd	GCTGTGGGAAAGTCAATGA
			rev	GCCATAGTAGTTTTCTAGGTATT
Mybl1	Myb proto-oncogene like 1	NM_008652.2	fwd	CCTAGCACTGAGTTTGGGGG
			rev	GGGAGTACTTCTGATGATGGATAC
Nanog	Nanog homeobox	NM_001289828	fwd	AGAGGAAGGGCGAGGAGA
			rev	TTCTTGCTTACAAGGGTCTGC
eNos	Nitric oxide synthase 3, endothelial cell	NM_008713	fwd	GCATGGGCAACTTGAAGA
			rev	AGGGTGTCGTAGGTGATG
iNos	Nitric oxide synthase 2, inducible	NM_010927	fwd	ACGAGACGGATAGGCAGAGA
			rev	GCACATGCAAGGAAGGGAAC
Nqo1	NAD(P)H quinone dehydrogenase 1	NM_008706.5	fwd	GGCCGATTCAGAGTGGCAT
			rev	CCAGACGGTTTCCAGACGTT
Nrf2	Nuclear factor erythroid 2-related factor 2	NM_001399226.1	fwd	CCCAGCAGGACATGGATTTGA
			rev	AGCTCATAGTCCTTCTGTCGC
Ocln	Tight junction protein occludin	NM_001360536.1	fwd	GTGAATGGGTCACCGAGGG
			rev	AAGATAAGCGAACCTGCCGAG
Oct2	Organic cation transporter-2	NM_001355767.1	fwd	TCGTCACTGAGTTTAACCT
			rev	AGAGATAGCATTGATGAGGAT
Oct4	Octamer-binding transcription factor 4	NM_001252452	fwd	CTGCCCCCAGGTCCCCACTT
			rev	AGCATCCCCAGGGAGGGCTG
Pecam1	Platelet endothelialcell adhesion molecule 1	NM_001032378	fwd	GCTCTGGGAACGAGAGCCACAG
			rev	CTGCTTTCGGTGGGGACAGGC
Pgc1α	Peroxisome proliferative activated receptor, gamma, coactivator 1 alpha	NM_008904	fwd	CCGAGAATTCATGGAGCAAT
			rev	TTTCTGTGGGTTTGGTGTGA
Rad51	RAD51 homolog (S. cerevisiae)	NM_011234	fwd	CAGCGATGTCCTAGATAATGTAG
			rev	TTACCACTGCGACACCAA
Slc7a11	Cystine/Glutamate Transporter	NM_011990.2	fwd	ATCTCCCCCAAGGGCATACT
			rev	GCATAGGACAGGGCTCCAAA
Sod1	Superoxide dismutase 1, soluble	NM_011434	fwd	ACCAGTTGTGTTGTCAGG
			rev	TTTCTTAGAGTGAGGATTAAAATGAG
Sod2	Superoxide dismutase 2, mitochondrial	NM_013671.3	fwd	GAATGTAATCAACTGGGAGAAT
			rev	CATAGAATTATCAGGTATGTGGAA
Tie1	Tyrosine kinase with immunoglobulin-like and	NM_011587	fwd	CAATGGGCCGTCTCCCTGTGC
			rev	GCGTGCCTCCAAGGCTCACT
Tjp1	Tight junction protein ZO-1	NM_001163574	fwd	CCCGGACTTTTGTCCCACTT
			rev	CCATTGCTGTGCTCTTAGCG
Top2a	Topoisomerase 2 alpha	NM_011623	fwd	CTTCAGGAGCCGTCACCAT
			rev	GAGCAGTATATGTTCCAGTTGT
Top2b	Topoisomerase 2 beta	NM_009409	fwd	TGGGTGAACAATGCTACAAA
			rev	TGTATGTATCAGGACGAAGGA
Tp53	Tumor protein p53	NM_011640	fwd	AAGTTCTGTAGCTTCAGTTCAT
			rev	GGCAGTCATCCAGTCTTC
Vcam-1		NM_011693	fwd	ATATACTTGGAAGTGTCGTGTT

	Vascular cell adhesion molecule 1		rev	GACCATCTTCACAGGCATT
VE-cad	Vascular endothelial cadherin	NM_009868	fwd	AAGGCTCAGCGCAGCATCGG
			rev	TCGTTGGCCTCCACAGTCAGGT

Supplementary table 2: Differentiation-dependent and drug-induced alterations in mRNA expression.

Summarized data shown are obtained from analysis of RT-qPCR-based mRNA expression analyses as presented in Fig. 1C, 2C and D, Fig. 5A-C, Fig. 6C, D and F, Fig. 7A-C and E, Fig. 8C, D and F.

Figure 1C

Fold of mESC (± SEM)						
	**Oct4**	**Nanog**	**Flk1**	**VE-cad**	**Tie1**	**Pecam1**
**EC d4**	0.02 *± 0.01*	0.03 *± 0.01*	15.47 *± 0.81*	467.55 *± 18.99*	64.36 *± 5.77*	0.60 *± 0.04*
**EC d4 + Dox 0.005 µM**	0.02 *± 0.01*	0.03 *± 0.01*	18.23 *± 1.04*	653.73 *± 41.28*	90.91 *± 6.69*	0.78 *± 0.04*
**EC d4 + Dox 0.05 µM**	0.02 *± 0.01*	0.02 *± 0.01*	26.95 *± 1.05*	1297.80 *± 104.71*	146.88 *± 20.36*	1.42 *± 0.06*
**EC d4 + B02 15 µM**	0.01 *± 0.01*	0.03 *± 0.01*	6.99 *± 0.32*	484.74 *± 24.38*	29.11 *± 0.86*	0.62 *± 0.03*
**EC d4 + EST 1 µM**	0.05 *± 0.01*	0.05 *± 0.01*	19.60 *± 1.34*	858.76 *± 29.87*	110.61 *± 8.58*	1.30 *± 0.06*

Figure 2C

Fold of mESC (± SEM)						
	**Atp7a**	**Ctr2**	**Oct2**	**Atp7b**	**Mdr1**	**Mrp1**
**EC d4**	0.59 *± 0.06*	2.05 *± 0.13*	0.09 *± 0.03*	0.83 *± 0.08*	2.58 *± 0.65*	0.96 *± 0.11*
**EC d6**	4.10 *± 1.63*	2.70 *± 1.21*	1.62 *± 1.04*	0.80 *± 0.26*	1.50 *± 0.64*	1.25 *± 0.54*

Figure 2D

Fold of control (± SEM)						
	**Atp7a**	**Ctr2**	**Oct2**	**Atp7b**	**Mdr1**	**Mrp1**
**mESC + Dox 0.05 µM**	2.50 *± 0.29*	2.95 *± 0.35*	5.33 *± 4.20*	1.82 *± 0.31*	4.03 *± 0.65*	2.38 *± 0.31*
**mESC + Dox 0.1 µM**	3.09 *± 0.56*	4.05 *± 0.69*	0.75 *± 0.25*	2.42 *± 0.57*	6.26 *± 1.24*	2.51 *± 0.44*
**EC d4 + Dox 0.005 µM**	1.11 *± 0.09*	1.25 *± 0.16*	0.33 *± 0.04*	1.04 *± 0.10*	1.46 *± 0.28*	1.32 *± 0.09*
**EC d4 + Dox 0.05 µM**	1.81 *± 0.20*	1.38 *± 0.10*	0.53 *± 0.06*	0.54 *± 0.07*	105.13 *± 6.29*	1.43 *± 0.11*
**EC d6 + Dox 0.05 µM**	1.47 *± 0.31*	1.13 *± 0.38*	0.25 *± 0.03*	0.99 *± 0.2*	4.89 *± 0.43*	1.23 *± 0.18*
**EC d6 + Dox 0.1 µM**	1.77 *± 0.40*	1.27 *± 0.43*	1.39 *± 1.77*	1.11 *± 0.06*	11.55 *± 0.95*	1.40 *± 0.10*

Figure 5A

Fold of mESC (± SEM)		
	**Top2a**	**Top2b**
**EC d4**	0.57 ± *0.09*	1.26 ± *0.14*
**EC d6**	1.10 ± *0.20*	3.25 ± *0.37*

Figure 5B

Fold of mESC (± SEM)					
	**Hmox1**	**Nos2**	**Nos3**	**Nrf2**	**Nqo1**
**EC d4**	0.40 ± *0.03*	0.74 ± *0.11*	0.01 ± *0.01*	0.38 ± *0.03*	0.19 ± *0.02*
**EC d6**	0.34 ± *0.04*	0.46 ± *0.04*	1.51 ± *0.26*	0.90 ± *0.09*	0.08 ± *0.01*

Figure 5C

Fold of mESC (± SEM)			
	**Brca1**	**Chk1**	**Rad51**
**mESC + Dox 0.1 µM**	1.93 ± *0.22*	0.68 ± *0.08*	1.36 ± *0.21*
**mESC + B02 20 µM**	0.95 ± *0.15*	1.33 ± *0.12*	0.90 ± *0.12*
**mESC + EST 20 µM**	0.52 ± *0.07*	0.27 ± *0.03*	0.29 ± *0.03*
**EC d4**	0.36 ± *0.06*	0.89 ± *0.13*	0.25 ± *0.04*
**EC d4 + Dox 0.05 µM**	0.25 ± *0.05*	0.34 ± *0.10*	0.27 ± *0.08*
**EC d4 + B02 15 µM**	0.27 ± *0.05*	0.92 ± *0.23*	0.19 ± *0.04*
**EC d4 + EST 1 µM**	0.32 ± *0.08*	0.51 ± *0.12*	0.26 ± *0.06*
**EC d6**	0.37 ± *0.11*	0.60 ± *0.18*	0.33 ± *0.10*
**EC d6 + Dox 0.1 µM**	0.45 ± *0.09*	0.43 ± *0.14*	0.48 ± *0.13*
**EC d6 + B02 20 µM**	0.51 ± *0.14*	1.27 ± *0.40*	0.34 ± *0.08*
**EC d6 + EST 20 µM**	0.19 ± *0.08*	0.44 ± *0.12*	0.11 ± *0.04*

Figure 6C

Fold of untreated control (± SEM)							
	**Gpx1**	**Gpx4**	**Gstm1**	**Hmox1**	**Pgc1a**	**Sod1**	**Sod2**
**mESC + Dox 0.05 µM**	1.54 *± 0.12*	0.62 *± 0.11*	2.08 *± 0.36*	1.14 *± 0.07*	1.78 *± 0.50*	1.30 *± 0.17*	1.10 *± 0.09*
**EC d6 + Dox 0.05 µM**	1.10 *± 0.05*	1.68 *± 0.08*	2.52 *± 0.20*	2.63 *± 0.19*	10.96 *± 2.59*	2.60 *± 0.42*	1.17 *± 0.06*

Figure 6D

Fold of untreated control (± SEM)	
	**Pgc1a**
**mESC + Dox 0.1 µM**	10.69 *± 4.79*
**mESC + B02 20 µM**	2.40 *± 0.70*
**mESC + EST 20 µM**	184.58 *± 44.52*
**EC d4 + Dox 0.05 µM**	24.33 *± 7.65*
**EC d4 + B02 15 µM**	0.11 *± 0.13*
**EC d4 + EST 1 µM**	0.21 *± 0.26*
**EC d6 + Dox 0.1 µM**	21.56 *± 4.13*
**EC d6 + B02 20 µM**	0.27 *± 0.38*
**EC d6 + EST 20 µM**	5.75 *± 1.86*

Figure 6F

Fold of untreated control (± SEM)		
	**Gpx4**	**Slc7a11**
**mESC + Dox 0.05 µM**	1.03 *± 0.09*	0.99 *± 0.07*
**mESC + Dox 0.1 µM**	1.05 *± 0.02*	1.20 *± 0.09*
**EC d4 + Dox 0.005 µM**	1.07 *± 0.07*	1.71 *± 0.12*
**EC d4 + Dox 0.05 µM**	1.35 *± 0.14*	3.43 *± 0.68*
**EC d6 + Dox 0.05 µM**	1.01 *± 0.06*	0.82 *± 0.10*
**EC d6 + Dox 0.1 µM**	0.99 *± 0.05*	1.09 *± 0.10*

Figure 7A

Fold of untreated control (± SEM)						
	**Il6**	**Il8**	**Cdkn2a**	**Cdkn1a**	**Tp53**	**Glb1**
**mESC + Dox 0.05 µM**	2.74 *± 0.48*	1.29 *± 0.12*	2.02 *± 0.16*	2.20 *± 0.20*	0.86 *± 0.10*	1.22 *± 0.10*
**mESC + Dox 0.1 µM**	2.84 *± 0.47*	1.07 *± 0.14*	2.49 *± 0.24*	2.09 *± 0.14*	0.87 *± 0.04*	1.35 *± 0.10*
**mESC + B02 10 µM**	0.98 *± 0.25*	0.76 *± 0.05*	0.97 *± 0.08*	0.89 *± 0.06*	1.09 *± 0.02*	1.06 *± 0.04*
**mESC + B02 20 µM**	1.35 *± 0.26*	0.93 *± 0.07*	0.89 *± 0.06*	0.94 *± 0.06*	0.98 *± 0.11*	1.06 *± 0.07*
**mESC + EST 10 µM**	6.25 *± 1.10*	0.83 *± 0.10*	5.82 *± 0.24*	2.08 *± 0.15*	0.83 *± 0.04*	0.47 *± 0.03*

Figure 7B

Fold of untreated control (± SEM)						
	**Il6**	**Il8**	**Cdkn2a**	**Cdkn1a**	**Tp53**	**Glb1**
**EC d4 + Dox 0.005 µM**	0.95 *± 0.31*	1.26 *± 0.44*	1.12 *± 0.46*	1.42 *± 0.60*	0.98 *± 0.39*	0.97 *± 0.25*
**EC d4 + Dox 0.05 µM**	1.91 *± 0.60*	25.17 *± 8.60*	3.09 *± 0.75*	4.89 *± 1.14*	1.48 *± 0.34*	1.54 *± 0.29*
**EC d4 + B02 5 µM**	0.80 *± 0.25*	0.83 *± 0.29*	1.24 *± 0.57*	1.71 *± 0.79*	0.98 *± 0.40*	1.45 *± 0.39*
**EC d4 + B02 15 µM**	1.35 *± 0.39*	0.85 *± 0.27*	1.39 *± 0.61*	2.57 *± 1.14*	0.72 *± 0.25*	2.55 *± 0.62*
**EC d4 + EST 0.5 µM**	0.99 *± 0.33*	0.80 *± 0.25*	1.29 *± 0.61*	1.59 *± 0.75*	1.018 *± 0.45*	1.41 *± 0.46*
**EC d4 + EST 1 µM**	1.09 *± 0.28*	0.57 *± 0.20*	1.49 *± 0.72*	1.62 *± 0.78*	0.99 *± 0.42*	1.61 *± 0.48*

Figure 7C

Fold of untreated control (± SEM)						
	**Il6**	**Il8**	**Cdkn2a**	**Cdkn1a**	**Tp53**	**Glb1**
**EC d6 + Dox 0.05 µM**	1.91 *± 0.65*	1.31 *± 0.30*	1.77 *± 0.18*	4.38 *± 0.34*	1.15 *± 0.08*	1.49 *± 0.09*
**EC d6 + Dox 0.1 µM**	3.23 *± 0.73*	1.15 *± 0.15*	2.66 *± 0.38*	5.67 *± 0.60*	1.09 *± 0.09*	1.97 *± 0.20*
**EC d6 + B02 10 µM**	0.85 *± 0.18*	0.52 *± 0.10*	0.64 *± 0.15*	1.67 *± 0.23*	0.97 *± 0.12*	0.88 *± 0.10*
**EC d6 + B02 20 µM**	1.79 *± 0.37*	0.32 *± 0.03*	1.15 *± 0.21*	2.28 *± 0.11*	1.27 *± 0.08*	1.64 *± 0.18*
**EC d6 + EST 10 µM**	1.19 *± 0.24*	0.24 *± 0.05*	1.85 *± 0.15*	1.91 *± 0.17*	0.82 *± 0.05*	3.60 *± 0.26*
**EC d6 + EST 20 µM**	2.47 *± 0.92*	0.48 *± 0.06*	2.02 *± 0.16*	2.49 *± 0.27*	0.96 *± 0.09*	5.40 *± 0.68*

Figure 7E

Fold of untreated control (± SEM)	
	**Dox 1 µM**
**Il6**	16.90<br>*± 0.86*
**Il8**	2.54<br>*± 0.25*
**Cdkn2a**	2.56<br>*± 0.14*
**Cdkn1a**	12.45<br>*± 0.91*
**Tp53**	1.66<br>*± 0.10*
**Glb1**	2.10<br>*± 0.11*
**Mmp3**	1.42<br>*± 0.09*
**Cxc1**	10.16<br>*± 1.13*
**E2f1**	0.54<br>*± 0.08*
**Mybl2**	0.053<br>*± 0.01*
**Ccn1a**	0.52<br>*± 0.12*
**Ccnd2**	2.92<br>*± 0.17*

Figure 8C

Fold of EC d4 or d6 (± SEM)			
	**Tjp1**	**Cldn5**	**Ocln**
**EC d4 + Dox 0.05 µM**	2.10 *± 0.30*	1.35 *± 0.32*	1.78 *± 0.40*
**EC d4 + B02 15 µM**	1.26 *± 0.16*	0.18 *± 0.01*	1.16 *± 0.20*
**EC d4 + EST 1 µM**	1.56 *± 0.17*	0.55 *± 0.04*	1.06 *± 0.16*
**EC d6 + Dox 0.1 µM**	1.21 *± 0.22*	2.50 *± 1.97*	1.79 *± 0.21*
**EC d6 + B02 20 µM**	1.07 *± 0.22*	4.85 *± 5.53*	1.87 *± 0.34*
**EC d6 + EST 20 µM**	1.46 *± 0.15*	2.16 *± 1.75*	1.62 *± 0.28*

Figure 8D

Fold of untreated control (± SEM)						
	**E-selectin**	**Icam-1**	**Vcam-1**	**iNos**	**eNos**	**Ccl2**
**EC d4 ctrl + I/T**	28.56 *± 4.20*	7.97 *± 0.55*	1.72 *± 0.10*	1.51 *± 0.06*	15.41 *± 1.30*	81.94 *± 5.73*
**EC d4 + Dox 0.005 µM**	58.37 *± 12.83*	3.06 *± 0.35*	0.44 *± 0.12*	4.75 *± 0.56*	135.52 *± 24.87*	2.40 *± 0.45*
**EC d4 + Dox 0.005 µM + I/T**	139.01 *± 36.11*	14.94 *± 2.31*	1.13 *± 0.27*	4.95 *± 0.85*	157.09 *± 35.76*	242.22 *± 37.61*
**EC d4 + Dox 0.05 µM**	485.89 *± 43.03*	53.37 *± 5.73*	0.085 *± 0.02*	3.20 *± 0.70*	239.30 *± 32.73*	23.54 *± 3.78*
**EC d4 + Dox 0.05 µM + I/T**	929.38 *± 73.50*	154.74 *± 16.32*	0.39 *± 0.02*	3.41 *± 0.65*	234.32 *± 43.07*	2314.32 *± 263.79*
**EC d4 + B02 5 µM**	120.46 *± 27.53*	3.12 *± 0.88*	0.19 *± 0.11*	2.01 *± 0.63*	153.40 *± 35.09*	2.96 *± 1.68*
**EC d4 + B02 5 µM + I/T**	257.65 *± 43.49*	9.69 *± 3.18*	0.36 *± 0.21*	2.26 *± 0.65*	184.04 *± 40.33*	279.77 *± 81.86*
**EC d4 + B02 15 µM**	126.11 *± 22.16*	1.02 *± 0.08*	0.08 *± 0.03*	0.43 *± 0.05*	47.93 *± 5.35*	0.38 *± 0.47*
**EC d4 + B02 15 µM + I/T**	165.16 *± 9.25*	2.52 *± 0.12*	0.12 *± 0.01*	0.33 *± 0.06*	55.08 *± 0.67*	84.86 *± 4.36*
**EC d4 + EST 0.5 µM**	45.82 *± 10.25*	6.04 *± 1.22*	0.80 *± 0.16*	4.21 *± 0.62*	156.02 *± 33.92*	4.20 *± 1.80*
**EC d4 + EST 0.5 µM + I/T**	121.29 *± 21.77*	26.69 *± 6.68*	1.68 *± 0.40*	4.06 *± 0.53*	165.80 *± 32.43*	454.50 *± 106.25*
**EC d4 + EST 1 µM**	24.24 *± 6.72*	5.21 *± 0.21*	0.49 *± 0.02*	1.14 *± 0.25*	58.94 *± 5.48*	0.90 *± 0.42*
**EC d4 + EST 1 µM + I/T**	66.25 *± 8.58*	19.80 *± 1.15*	1.55 *± 0.09*	1.30 *± 0.41*	53.06 *± 5.05*	132.23 *± 18.49*

Figure 8F

Fold of untreated control (± SEM)	
	**Ldlr**
**Dox 0.05 µM**	0.45 *± 0.04*
**B02 15 µM**	1.78 *± 0.08*
**EST 1 µM**	0.66 *± 0.05*

Supplementary table 3: Differentiation stage dependent drug-induced changes in protein expression.

Summarized data shown below are obtained from densitometrical analyses of western blot-based protein expression analyses as depicted in Fig. 1D, 4H, 5D and 6E.

Fig. 1D

Fold of mESC	mESC				EC d4				EC d6			
	Ctrl	Dox	B02	EST	Ctrl	Dox	B02	EST	Ctrl	Dox	B02	EST
H4ac	1.0	0.0	232.9	7426.8	0.0	0.0	0.0	1853.2	0.00	0.0	0.0	4083.7
H3ac	1.0	0.3	1.8	16.6	0.1	0.0	0.0	4.6	0.3	0.0	0.1	7.8
αTubac	1.0	0.5	0.5	1.0	1.2	0.3	0.6	0.9	1.4	0.6	0.3	1.0

Fig. 4H

Fold of mESC	mESC					
	Ctrl	Eto	Dox 1	Dox 2	B02	EST
p-p53	1.0	6.1	6.4	6.5	1.2	0.9
p-ATR	1.0	1.6	1.9	2.7	2.7	2.9
p-CHK1	1.0	2.3	2.4	4.4	0.8	0.1
CHK1	1.0	1.0	1.0	1.3	1.4	1.0
RAD51	-	-	-	-	-	-
p-RPA32	1.0	1.2	1.1	1.5	2.0	1.3

Fig 5D - Dox

Fold of mESC	mESC				EC d4				EC d6			
	Ctrl	Eto	Dox 1	Dox 2	Ctrl	Eto	Dox 1	Dox 2	Ctrl	Eto	Dox 1	Dox 2
γH2AX	1.0	186.9	69.7	174.9	89.4	244.6	142.6	44.9	0.0	1.1	4.6	31.8
p-p53	1.0	6.9	4.2	5.7	2.5	4.8	2.0	2.6	1.3	3.7	4.2	3.1
p-KAP1	1.0	29.7	31.2	59.9	0.0	0.0	0.0	0.0	0.0	0.0	0.0	0.0
p-CHK2	1.0	1.6	1.1	1.5	0.7	0.5	0.7	0.2	0.4	0.2	0.8	0.5
p-RPA32	1.0	1.6	1.4	1.8	0.6	0.5	0.5	0.1	0.5	0.1	0.2	0.1
RAD51	1.0	1.9	1.2	0.9	0.4	0.1	0.1	<0.1	0.7	0.8	1.7	1.3
CHK1	1.0	2.4	1.7	1.8	1.8	1.6	2.1	0.2	1.9	0.6	1.7	1.0

Fig. 5D - B02

Fold of mESC	mESC				EC d4				EC d6			
	Ctrl	Eto	B02 1	B02 2	Ctrl	Eto	B02 1	B02 2	Ctrl	Eto	B02 1	B02 2
γH2AX	1.0	5.6	<0.1	0.5	1.2	2.5	1.8	0.0	1.2	0.38	1.2	0.1
p-p53	1.0	2.1	0.9	1.2	0.7	1.7	0.6	0.3	0.4	1.45	0.4	0.2
p-KAP1	1.0	49.01	1.30	1.26	0.0	0.0	0.0	0.0	0.0	0.00	0.0	0.0
p-CHK2	1.0	0.8	0.9	1.1	0.2	0.4	0.2	<0.1	0.1	0.04	0.1	0.0
p-RPA32	1.0	0.7	0.8	1.3	0.4	0.4	0.3	0.4	0.3	0.04	0.1	<0.1
RAD51	1.0	1.9	1.9	2.3	0.6	1.0	0.7	0.3	0.6	0.38	0.7	<0.1
CHK1	1.0	0.9	0.8	1.0	0.5	0.7	0.4	0.2	0.6	0.37	0.7	0.1

Fig. 5D – EST

Fold of mESC	mESC				EC d4				EC d6			
	Ctrl	Eto	EST 1	EST 2	Ctrl	Eto	EST 1	EST 2	Ctrl	Eto	EST 1	EST 2
γH2AX	1.0	6.2	7.7	10.1	3.3	12.2	9.1	8.9	0.3	0.0	1.3	0.1
p-p53	1.0	3.7	0.9	0.8	0.8	3.0	0.4	0.5	0.4	2.0	0.3	0.3
p-KAP1	1.0	36.8	2.9	8.2	0.0	0.0	0.0	0.0	0.0	0.0	0.0	0.0
p-CHK2	1.0	1.3	1.0	1.0	1.1	1.2	0.6	0.9	0.7	0.4	0.5	0.3
p-RPA32	1.0	1.0	0.7	0.7	0.4	0.4	0.8	0.9	0.4	<0.1	<0.1	<0.1
RAD51	1.0	2.4	1.4	1.4	1.4	1.2	1.0	0.9	0.7	0.5	0.6	0.1
CHK1	1.0	1.3	0.8	0.7	1.2	0.7	0.7	1.0	0.8	0.1	0.6	0.2

Fig 6E

Fold of mESC	mESC				EC d4				EC d6			
	Ctrl	Dox	B02	EST	Ctrl	Dox	B02	EST	Ctrl	Dox	B02	EST
p-p70SK	1.0	1.1	1.2	0.8	0.8	0.5	1.4	1.6	1.0	2.8	1.1	0.7
AMPKa	1.0	0.2	0.2	0.5	0.2	0.7	0.1	0.5	0.1	0.6	0.1	0.7
GSK3b	1.0	0.8	1.3	1.2	2.4	5.7	1.9	2.3	2.1	4.3	1.8	1.7
